# Processive Enzymes Kept on a Leash: How Cellulase Activity in Multienzyme Complexes Directs Nanoscale Deconstruction of Cellulose

**DOI:** 10.1021/acscatal.1c03465

**Published:** 2021-10-25

**Authors:** Krisztina Zajki-Zechmeister, Gaurav Singh Kaira, Manuel Eibinger, Klara Seelich, Bernd Nidetzky

**Affiliations:** † Institute of Biotechnology and Biochemical Engineering, Graz University of Technology, Petersgasse 10-12/1, 8010 Graz, Austria; ‡ Austrian Centre of Industrial Biotechnology, Petersgasse 14, 8010 Graz, Austria

**Keywords:** polymer deconstruction, cellulose, complexed and noncomplexed cellulases, enzyme assembly, enzyme synergy, processive depolymerization

## Abstract

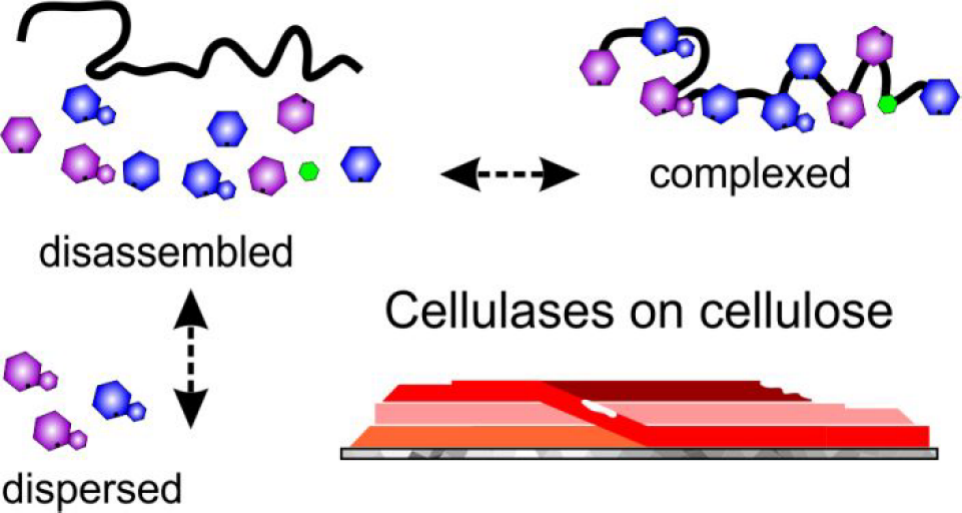

Biological deconstruction of polymer materials gains efficiency from the spatiotemporally coordinated action of enzymes with synergetic function in polymer chain depolymerization. To perpetuate enzyme synergy on a solid substrate undergoing deconstruction, the overall attack must alternate between focusing the individual enzymes locally and dissipating them again to other surface sites. Natural cellulases working as multienzyme complexes assembled on a scaffold protein (the cellulosome) maximize the effect of local concentration yet restrain the dispersion of individual enzymes. Here, with evidence from real-time atomic force microscopy to track nanoscale deconstruction of single cellulose fibers, we show that the cellulosome forces the fiber degradation into the transversal direction, to produce smaller fragments from multiple local attacks (“cuts”). Noncomplexed enzymes, as in fungal cellulases or obtained by dissociating the cellulosome, release the confining force so that fiber degradation proceeds laterally, observed as directed ablation of surface fibrils and leading to whole fiber “thinning”. Processive cellulases that are enabled to freely disperse evoke the lateral degradation and determine its efficiency. Our results suggest that among natural cellulases, the dispersed enzymes are more generally and globally effective in depolymerization, while the cellulosome represents a specialized, fiber-fragmenting machinery.

## Introduction

Biochemical upgrading of polymer materials in natural residues and polluting wastes represents a central element of the transition to a sustainable bioeconomy.^1−4^ Upgrading usually involves, as a first step, the deconstruction of the solid polymer substrate via catalytic depolymerization of the polymer chains into the soluble monomers.^5−8^ By virtue of its selectivity and the benign conditions used, biological (enzyme-catalyzed) depolymerization is widely regarded as the preferred option for material deconstruction, as opposed to alternative(s) from pure chemistry.^6^ Biological deconstruction relies on cooperative catalysis by multienzyme systems as a common mechanistic principle. The principle is exemplified on a large variety of biomaterials built from hydrolyzable polymer chains (e.g., polysaccharides,^9−11^ structural proteins,^12^ polyesters^11,13,12−15^). In all cases, the material deconstruction gains efficiency from the spatiotemporally coordinated action of different enzymes with synergetic function in polymer chain depolymerization.^10,16^ Typically, a set of chain end-cleaving and internally chain-cleaving hydrolases act in synergy.^9,10^ Even in xenobiotic materials such as polyethylene terephthalate, the naturally evolved enzyme system for biological deconstruction of the plastics exploits the synergy between two hydrolases.^13,14,16,17^

On soluble polymer substrates, there is hardly any structural restriction for enzymes to synergize. However, most polymer materials are solid. Their nanoscale morphology typically shows ordered (crystalline) phases interspersed with regions of irregular structure.^18−23^ Spatial irregularities result from orientational and directional disorder in polymer chain organization into solid material.^24−27^ Only a limited fraction of the enzyme-accessible external surface is usually available for polymer chain cleavage by the individual types of hydrolases.^28−30^ For an enzyme system confined to working at the surface of such solids, there thus arises the fundamental challenge of a locally focused usage of enzyme synergy. Moreover, there is the additional challenge of perpetuation of enzyme synergy as the solid surface evolves because of the substrate’s ongoing deconstruction. Generally, the overall enzyme attack would thus be expected to consist in dynamical cycles of local concentration and dispersion of the individual enzymes.^31^ The concentration benefits enzyme synergy momentarily, while dispersion is necessary to maintain it, by enabling the enzymes to access fresh “chain attack” sites on the surface.^30,32^ Besides desorption and readsorption, dispersion involves molecular diffusion as well as directed movement on the solid surface. As concentration and dispersion are opposing processes, their spatiotemporal coordination is crucial. Little is known about how enzyme systems steer the attack cycles of their individual synergetic activities for efficient interfacial (solid surface-directed) catalysis to substrate chain depolymerization.

The abundant biomaterial cellulose has been the center of attention for decades because of its enzymatic deconstruction into glucose and further upgrading into fuels and chemicals.^5,6,33^ Natural hydrolase systems for cellulose deconstruction (cellulases) reflect in their molecular organization the two opposing extremes of the concentration–dispersion cycle of synergetic enzyme attack on a solid substrate (Figure S1). The cellulosome, found in select anaerobic bacteria and fungi,^34,35^ is a large protein complex that has multiple enzymatic subunits assembled on a flexible scaffold protein (Figure S1A).^36,37^ The cellulases of cellulose-degrading fungi are ensembles of individual enzyme molecules (Figure S1B).^10^ In placing enzymes of synergetic function in close spatial proximity, the cellulosome maximizes the effect of local concentration.^36,38^ Complexation on the other hand restricts dispersion of the enzymes. The cellulase ensembles, in contrast, enable free dispersion of enzymes^39^ but lack the force from complexation to promote local concentration.^32^ The idea inspired by the cellulosome, that molecular proximity can leverage synergizing enzymes to improved efficiency, is supported from evidence on engineered enzyme complexes, the so-called designer cellulosomes,^40,41^ and multienzymatic fusion proteins.^42−45^ The assembled/covalently linked enzymes are generally reported as more active in substrate hydrolysis (cellulose^43,44,46,47^ but also other materials including plastics^17,48^) than the corresponding mixture of the disassembled/separated enzymatic units. However, with natural and engineered cellulase systems alike, we lack mechanistic understanding of how activity in multienzyme complexes directs the nanoscale deconstruction of the cellulosic substrate in comparison to activity of a dispersed ensemble of the same or relevantly similar enzymes. Only with this fundamental knowledge will it become possible to assess enzyme assembly into complexes with respect to intensifying the cellulose degradation. Deepened insight obtained for the cellulases may inform enzymatic degradation of solid substrates in general.

Here, we developed an advanced experimental framework to characterize cellulase synergetic activity in terms of the nanoscale deconstruction work done on solid material. The methodology solves a fundamental dilemma regarding the cellulose substrate applied in such an inquiry. The substrate should be representative of the fiber morphology of natural celluloses, yet it must feature a well-defined nanoarchitecture to enable continuous (real-time) tracking of enzymatically caused deconstruction events at high (∼1 nm) spatial resolution. Struggling with exactly this dilemma, earlier studies of related purport^49−51^ and our own^38,52−54^ were limited in the scope of observation and interpretation. Although instrumental to unravel the single-molecule behavior of certain cellulases, near-perfect crystallites^55,56^ of cellulose lack “amorphous domains”, that is, areas of organizational disorder of the polysaccharide chains.^19,21,22,27,57^ Such amorphous domains represent recurrent features of nanostructure distinctive of natural cellulose fibrils.^58,59^ The crystallites therefore represent only partial substrates for cellulase multienzyme systems and have limited purview for assessing synergy between individual enzyme types in substrate deconstruction. We show that single fibers (bundles of microfibrils) of bacterial cellulose,^60,61^ released carefully from the fiber network of the native material, met the challenging functional-analytical requirements of investigation comprehensively. Employing this dedicated substrate preparation in a combined atomic force microscopy (AFM) and biochemical study, we succeeded in obtaining evidence that clarifies the role of cellulase complexation in natural deconstruction.

## Results

### Single-Fiber Cellulose Substrate

**Figure 1:**
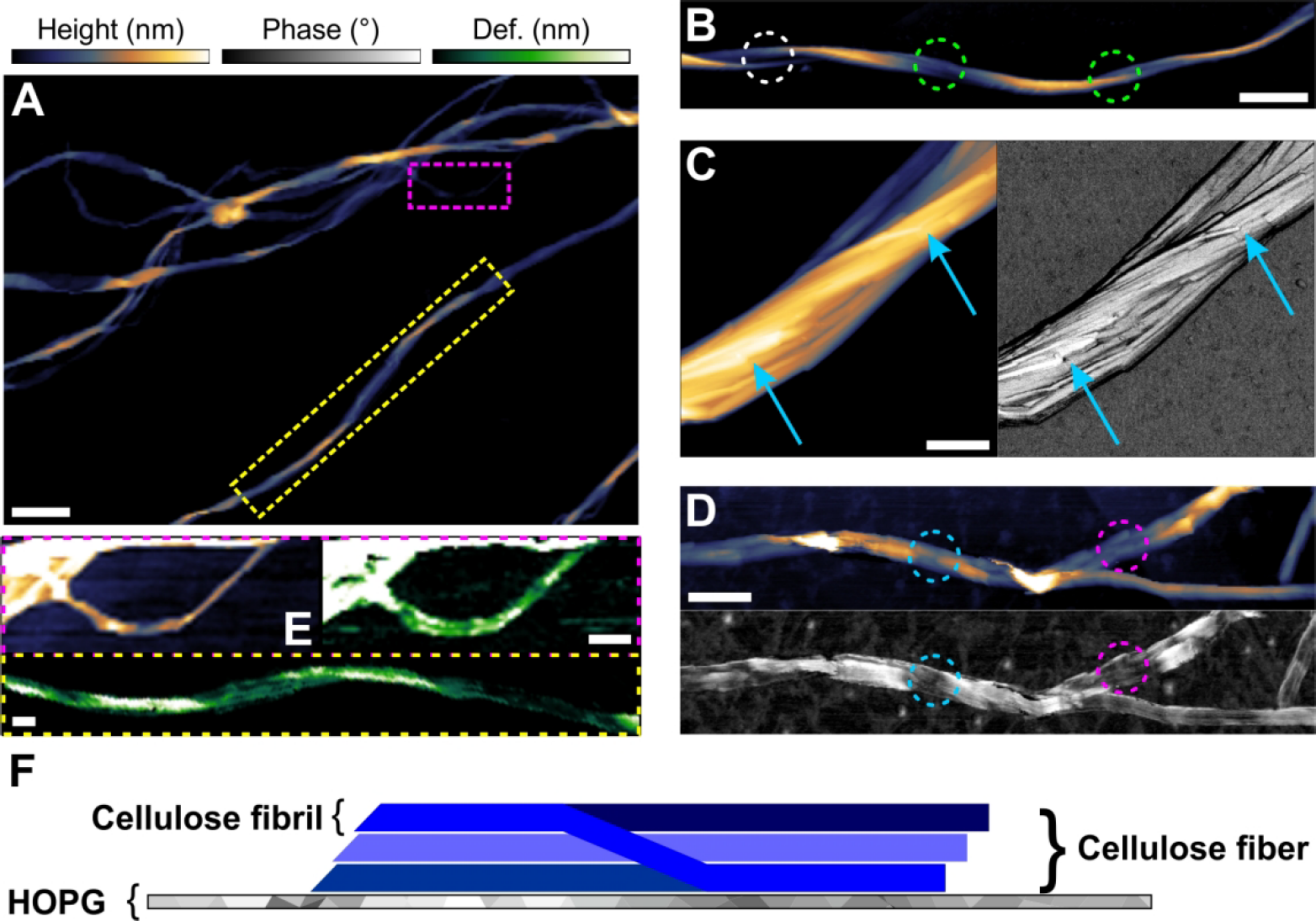
AFM characterization of bacterial cellulose. (A, B) AFM height image of (A) multiple fibers adsorbed on HOPG and (B) an isolated fiber bundle showing multiple twists (encircled in green) and an unwound region (encircled in white). An expanded view on the highlighted areas (colored rectangles) in panel A is shown in panel E. (C, D) AFM height (left) and phase images (right) of a close-up region of the fiber to reveal the fibrillar substructure. In panel C, some fibrils exhibit dislocations (lower-left blue arrow) or end internally (upper-right blue arrow). In panel D, a regular pattern of structural defects (less well-ordered regions of the cellulose, seen as darker areas in the phase images) is identified. These defects occur even in the absence of a discernible feature in the height channel (pink circle). Their regular spacing suggests structural connection with the fiber twist. (E) Peak force tapping also confirmed a somewhat regular alternating appearance of nanodomains with higher/lower deformation under force load. The upper left image is a zoomed and rescaled height image (taken from panel A) to allow an easier viewing. Note that calibration for peak force was performed on HOPG. (F) Schematic representation of a fiber adsorbed on HOPG. Scale bars are 250 nm (A, B) and 100 nm (C, D, E). The false color scales used throughout the images are shown in panel A. Height (nm), phase (deg), and deformation (nm) ranges were 42 nm (A), 45 nm (B), 80 nm/16° (C), 15 nm/24° (D), 10 nm/3 nm deformation (E, upper panel), and 15 nm deformation (E, lower panel).

Cellulose substrate pertinent to the mechanistic inquiry was prepared from bacterial (*Acetobacter xylinum*) cellulose. Single cellulose fibers were released by controlled ultrasonication from the three-dimensional network of fibers synthesized by the bacteria (Figure S2). For AFM analysis, an appropriately diluted suspension of the sonicated cellulose was adsorbed on the surface of highly oriented pyrolytic graphite (HOPG) (Figure 1A,D; Figure S2). The single fibers are ∼1 μm long bundles of regularly twisted (1–2 twists/μm) and strongly intertwined cellulose fibrils, as shown in Figure 1B. They exhibit a right-handed chirality (Figure S3), with this, a similarity in supramolecular chirality with plant-derived nanocelluloses is noted.^62^ Data from X-ray diffraction show the fibrils to be of high (≥90%) bulk crystallinity and to exhibit natural cellulose Iα crystal structure^60,63^ (Figure S4). The cellulose Iα crystallinity implies each microfibril contains chains oriented in parallel.^60^ We note that plant cellulose mostly exhibits Iβ crystallinity, featuring a slightly different interchain hydrogen bond pattern in parallel-oriented chains as compared with cellulose Iα.^56^ The crystal phases (Iα, Iβ) often occur together in variable ratio in natural celluloses.^10,64^ The crystal phase composition of cellulose will certainly affect the kinetic properties of the enzymes assayed with that substrate. However, fundamental characteristics of cellulase, for example, whether an enzyme cleaves chains internally or in a processive fashion, are typically not variably dependent on the substrate’s crystal structure.^65,66^ Therefore, while the distinction between cellulose Iα and Iβ is important, it is exceedingly improbable that the overall mode of nanoscale deconstruction of the cellulose fiber by a cellulase system would be critically determined by the type of crystal phase.

Using material-sensitive AFM measurements in tapping mode performed on sample in reaction buffer, we distinguish microfibril regions of differing nanomechanical properties.^67,68^ AFM results acquired in phase imaging (Figure 1C, D) and peak force tapping mode (Figure 1E) reveal nanodomains of higher and lower phase contrast and deformation, respectively, and show these domains to alternate in a somewhat regular fashion along the fibril length. The deformation reflects the material rigidity directly. The phase contrast serves as a general (compounded^69^) reporter of alterations in overall material character. Changes in the measured parameters are therefore good indications of a different organizational order of the cellulose chains. Nanodomains of low structural order are found primarily in regions of the microfibril affected by the internal twist (Figure 1C–E). Adopting terminology from the materials sciences, we refer to these nanodomains as “defects” in the molecular organization of the polymer.^70,71^ Overall, the single cellulose fibers used exhibit well-defined and reproducible features of structural nanoarchitecture at the levels of the individual microfibril and the microfibril bundle/fiber (Figure 1F).

In addition to their regular fibrillary structure, the cellulose fibers show deformations on a larger scale, like unwound regions where fibrils are separated from the bundle composite (Figure 1B, white circle) or internally ending fibrils which are typically seen as sudden and locally restricted decreases in height (Figure 1C, arrows). The deformations are arguably caused by the ultrasonication procedure used to isolate single fibers of the cellulose. Precisely because they can represent the effect of mechanical refining on crystalline cellulose, these deformations were interesting additional features to be analyzed for enzymatic deconstruction.^70,71^

### Cellulose Fiber Deconstruction by the Cellulosome

To study the activity of multienzyme complexes, we used the cellulosome purified from the supernatant of *Clostridium thermocellum* grown on cellulose. The *C. thermocellum* cellulosome is well-characterized^36,72^ and can be considered prototypical of this class of cellulases. In its full-fledged form, as obtained on average by the procedures used, the cellulosome comprises a scaffold protein (CipA) of nine subunits harboring a complete set of synergistically acting, carbohydrate-depolymerizing enzyme activities^36,73^ and a family 3 carbohydrate binding module. Note that CipA might be naturally integrated further into secondary scaffoldins (e.g., OlpB), forming even larger carbohydrate-depolymerizing entities comprising up to 63 individual enzymes.^36,74^

Using a tailored set up for real-time AFM measurements, images were recorded from the enzymatic reaction over a time span (∼3 h) and with a temporal resolution (∼0.05–0.50 frames/s) carefully adjusted to the rates of observable nanoscale deconstruction events. The data processing was automated and the analysis human user-independent by a self-programmed, dedicated MATLAB routine (Figure S5–S9). Full image sets are presented as movies (Supporting Information).

**Figure 2:**
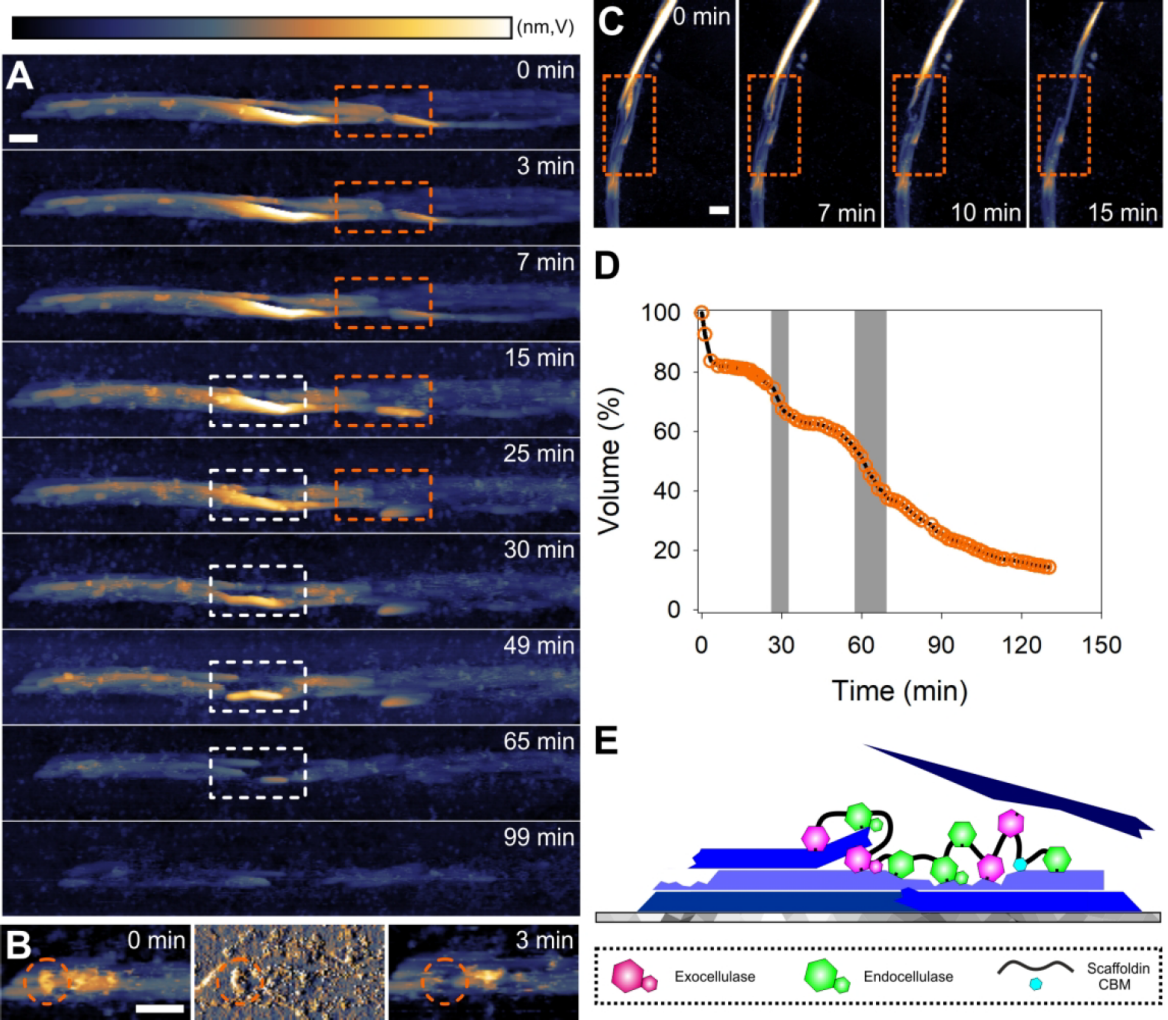
Cellulose fiber deconstruction by the cellulosome. (A) Time-lapse AFM height images taken from Movie S1 showing fiber deconstruction by the cellulosome to proceed via localized cuts into fibrils (framed in orange and white) that destabilize the overall fiber architecture and lead to whole fiber fragmentation. (B) AFM height (left, right) and amplitude (center) images taken from Movie S1 showing a single cellulosome (circled in orange) adsorbed on a cellulose fibril. After its dissociation, the cellulosome leaves behind a cavity (also circled in orange) due to the material removed underneath. (C) Time-lapse AFM height images taken from Movie S2 showing that fibrils attacked by cellulosomes can become “mobile” due to release from the fibril composite (indicated by a distorted shape, orange rectangle) and are subsequently removed by the AFM tip, with details shown in Movie S2. (D) Time course of volume loss during fiber degradation by the cellulosome (prominent steps are highlighted in gray). (E) Schematic representation of cellulosomal fiber deconstruction. Scale bars are 50 nm. The false color scale used throughout the images is shown in panel A. Height (nm) and amplitude (V) ranges were 30 nm (A), 15 nm/5 V (B), and 40 nm (C).

Viewed overall (Movie S1), the fiber deconstruction consists in multiple events of localized (∼100 nm longitudinal range) disruption of the fiber’s internal structure, eventually leading to fragmentation into smaller pieces (Figure 2A). Analyzed in more detail, the enzymatic attacks are focused on fiber regions featuring structural defects in multiple adjacent microfibrils or showing mechanically induced deformations (Figure 2A, B). Although single-molecule tracking was not pursued with these experiments, cellulosomes were seen frequently to be adsorbed stably (≥2 min) at the attacked sites (Figure 2B, Movies S1 and S2). The deconstruction begins with adjoining cuts at deformations of surface-exposed fibrils (Figure S10) to remove material over ∼30 nm of fibril length. Due to destabilization of the fibrillar organization in the regions affected, fibrils or parts thereof are released from the composite structure, overall visible as local fraying of the fiber (Figure 2A, C, Movies S1 and S2). Uppermost fibrils thus become more mobile and are occasionally moveable by the AFM tip during further scanning of the region, an effect that is not observed during fiber measurements without active enzymes present (Figure 2C, Figure S11 and Movie S2). This in combination with the ongoing enzymatic degradation causes the fiber to break apart completely. Parts of the fiber not affected by the fragmentation have hardly changed in height.

To track the fiber deconstruction on the basis of a quantifiable parameter, we determined the decrease in fiber volume dependent on reaction time. The cellulosome removes fiber volume in discrete steps (Figure 2D). This is consistent with a process of fiber splitting into progressively smaller fragments that are eventually fully degraded or removed from the graphite surface (Figure 2E, Movies S1 and S2).

### Cellulose Fiber Deconstruction by Noncomplexed (Dispersed) Cellulases

**Figure 3:**
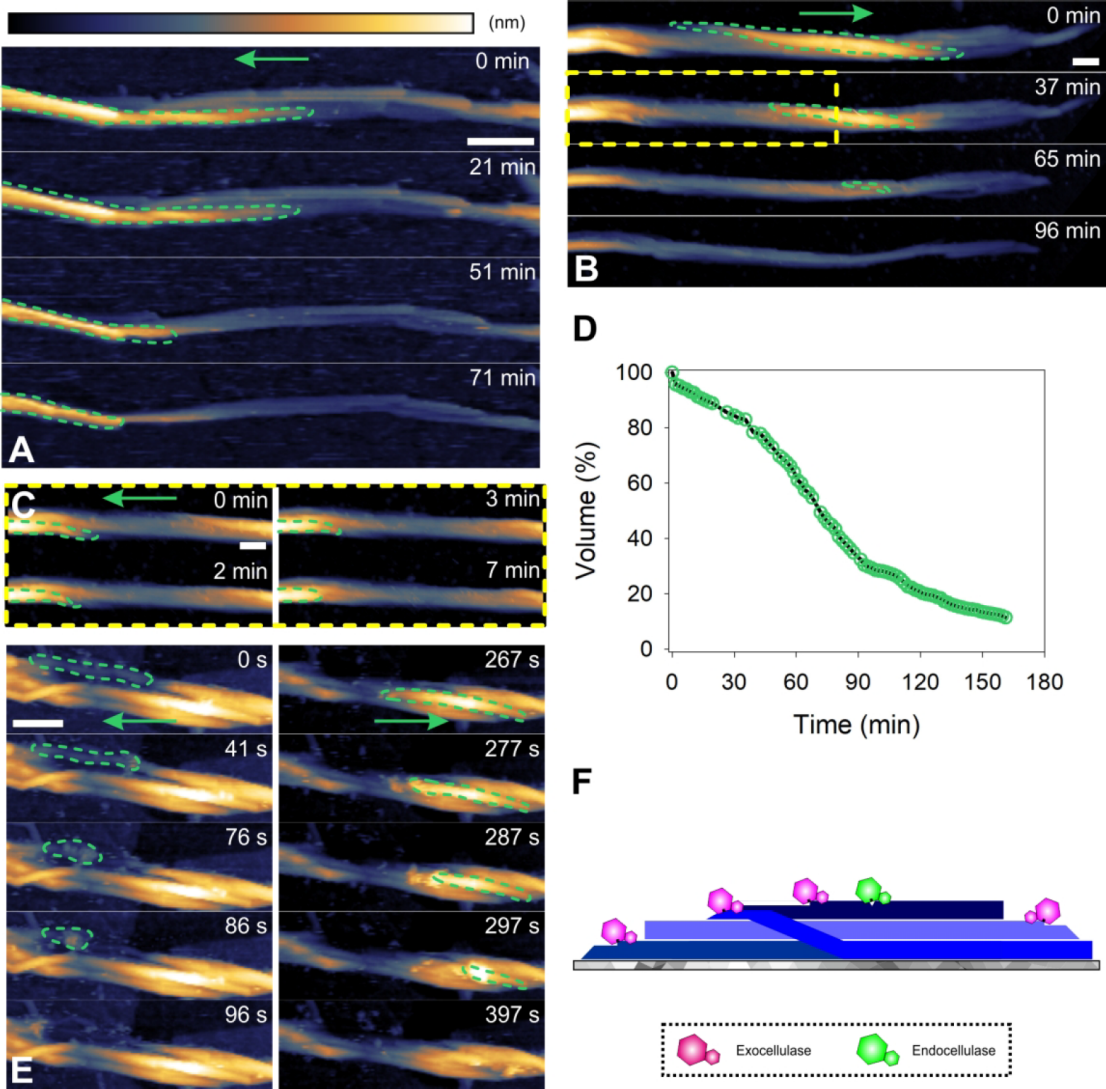
Cellulose fiber deconstruction by dispersed cellulases. (A, B, C) Time-lapse AFM height images taken from Movies S3 (A) and S4 (B, C) showing fiber deconstruction (green frame) by free cellulases to proceed via longitudinal thinning, due to directed ablation of surface fibrils (indicated with arrows). An expanded view on the highlighted area (yellow rectangle) in panel B is shown in panel C. Different fibrils from the same fiber in panels B and C are degraded in opposite direction. (D) Time course of volume loss during fiber degradation by dispersed cellulases. (E) The pattern, that individual fibrils are degraded unidirectionally but different fibrils can be degraded in opposite direction of the fiber’s length axis, remains in experiments with purified enzymes (Cel7A, Cel7B) but lacking the alternate cellobiohydrolase Cel6A. (F) Schematic representation of fiber deconstruction by dispersed cellulases. Scale bars are 50 nm. The false color scale used throughout the images is shown in panel A. Height (nm) range was 42 nm (A, B), 18 nm (C), and 18 nm (E).

The cellulase system of the fungus *Trichoderma reesei* was used.^5^ This cellulase is well-characterized and prototypical of the dispersed cellulase class.^5,75^ In sharp contrast to the cellulosome, the *T. reesei* cellulases degrade the cellulose fiber by longitudinal thinning (Figure 3A, Movie S3). Deconstruction consists in multiple events of directed ablation of fibrils from the dynamically evolving fiber surface (Figure 3A–C, Movies S3 and S4). While outer lying fibrils are gradually removed, the internal fiber structure remains largely intact and fiber fragmentation is minimal (Figure 3A–C, Movies S3 and S4). The removal of fiber volume with time is continuous, as shown in Figure 3D. Although the fibril ends were usually well accessible to the cellulases, the fibril ablation often starts internally at positions of defect in molecular organization of the cellulose chains (Movie S2). In terms of recognition of attack sites on the cellulose fibril, therefore, important similarity between cellulases and the cellulosome is noted. Interestingly, fiber parts showing larger structural deformation are not attacked faster, or with higher preference, than fibril-internal regions of defect.

Although each fibril is degraded unidirectionally by the cellulases,^55,76,77^ the direction of degradation changes between different fibrils (cf. Figure 3B, C, Movie S4). The finding is important mechanistically. We considered that besides its major cellobiohydrolase (Cel7A) that degrades cellulose chains from the reducing end,^10^ the *T. reesei* cellulase comprises another cellobiohydrolase (Cel6A) of opposite (nonreducing) chain-end specificity.^78^ Fiber deconstruction by isolated enzymes (i.e., Cel7A and the endoglucanase Cel7B) under exclusion of the Cel6A involves direction change in fibril degradation analogous to the native cellulase (Figure 3E). These results therefore identify fibril orientation in the fiber as the determinant of the observable direction of the enzymatic fibril degradation (Figure 3F). Their implication, that fibrils are assembled in the fiber in different (parallel as well as antiparallel) orientation, clarifies an elusive feature of the supramolecular organization of bacterial cellulose.^23^

### Cellulose Fiber Deconstruction by a Noncomplexed (Disassembled) Cellulosome

The immediate suggestion from the aggregate evidence on fiber deconstruction by the cellulases and the cellulosome is the following: (1) enzyme assembly into stable complexes forces substrate degradation through localized attacks in the transversal direction of the fiber; and (2) dispersed enzymes release the confining force so that substrate degradation proceeds laterally. To subject the implied hypothesis to a critical test, we dissociated the cellulosome’s enzymatic subunits from the scaffold protein^79^ and applied the resulting mixture of dispersed enzymes to AFM study of cellulose fiber construction. The individual subunit composition of the native cellulosome is retained largely in the dispersed preparation^80^ (Figure S12). The specific activity of soluble sugar release from the used cellulose is ∼3-fold less for the dispersed compared to the native cellulosome (see later). By way of the enzyme loading, the AFM experiments compensate this difference.

**Figure 4:**
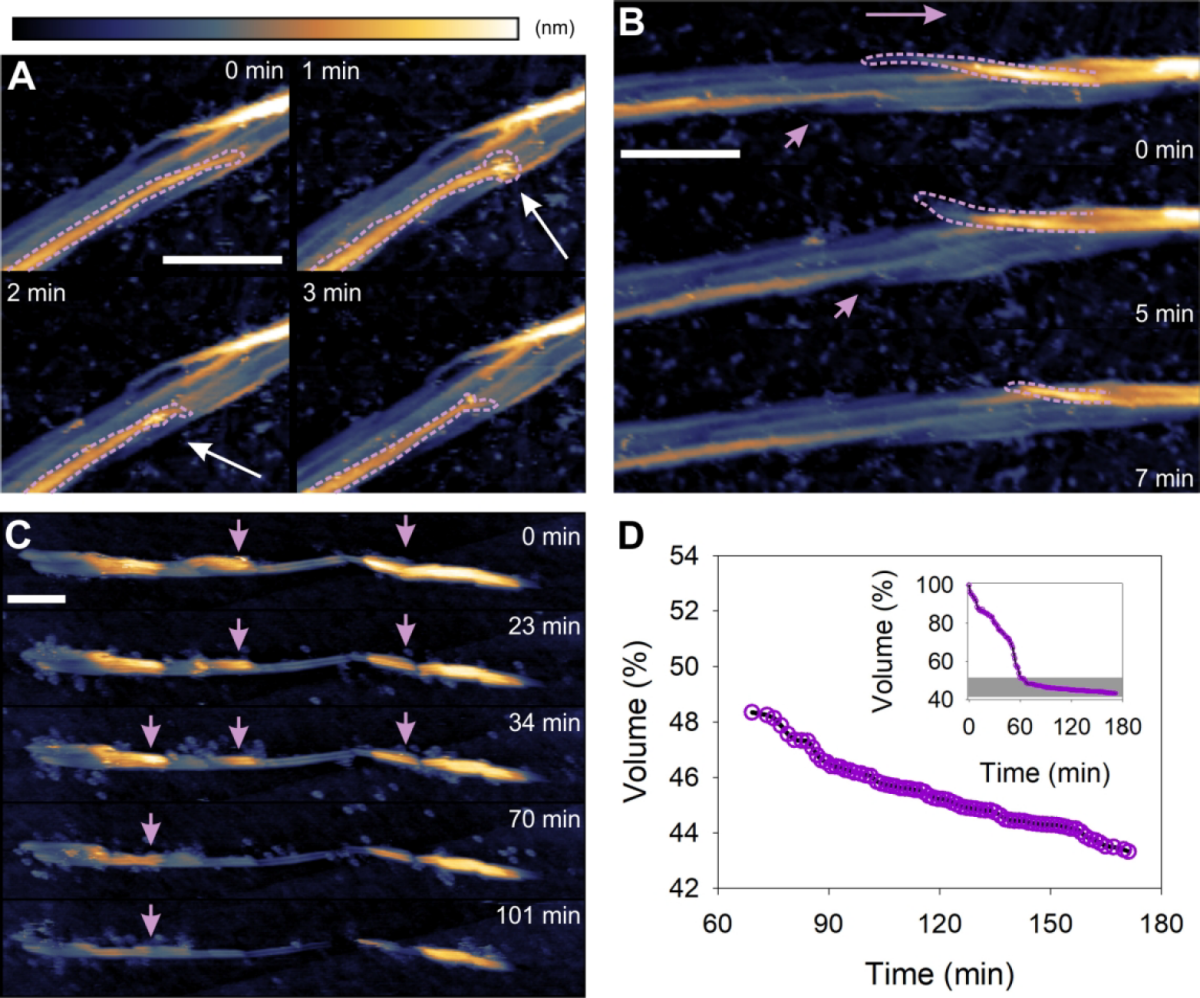
Cellulose fiber deconstruction by a noncomplexed (disassembled) cellulosome. (A, B, C) Time-lapse AFM height images taken from Movies S5 (A, B) and S6 (C) showing fiber deconstruction (purple frames/arrows) by disassembled cellulosomal cellulases to proceed similar as observed for naturally dispersed cellulases. Degradation occurs via ablation of surface fibrils (A, B) by mostly isolated enzymes (white arrow) and thinning/ablation (C). (D) Time course of volume loss during fiber degradation by the disassembled cellulosome. Note that the shown graph starts at about 60 min after an instable fiber on top is torn away (see Movie S6). The full time course is shown in the inset, and the presented time period is underlaid in gray. Scale bars are 100 nm. The false color scales used throughout the images is shown in panel A. Height (nm) range was 12 nm (A, C) and 14 nm (B).

Results (Movies S5 and S6, Figure 4) show that the dispersed cellulosomes behave in fiber deconstruction as predicted. Their mode of action is characteristically similar to that of the *T. reesei* cellulases, consisting in the mainly unidirectional ablation of single fibrils on the surface (Figure 4A, B) and leading to whole fiber thinning (Figure 4C). Change of degradation direction between different fibrils of one fiber can be observed again (cf. Figure 4A, B). Localized fragmentation of the cellulose fiber is not observed, indicating that the defining feature of the native cellulosome’s mode of substrate deconstruction has been lost. The dispersed cellulosomes remove fiber volume continuously, as implied by a substrate degradation that proceeds via ablative thinning (Figure 4D).

### Cellulose Hydrolysis by Dispersed and Complexed Cellulases

**Figure 5:**
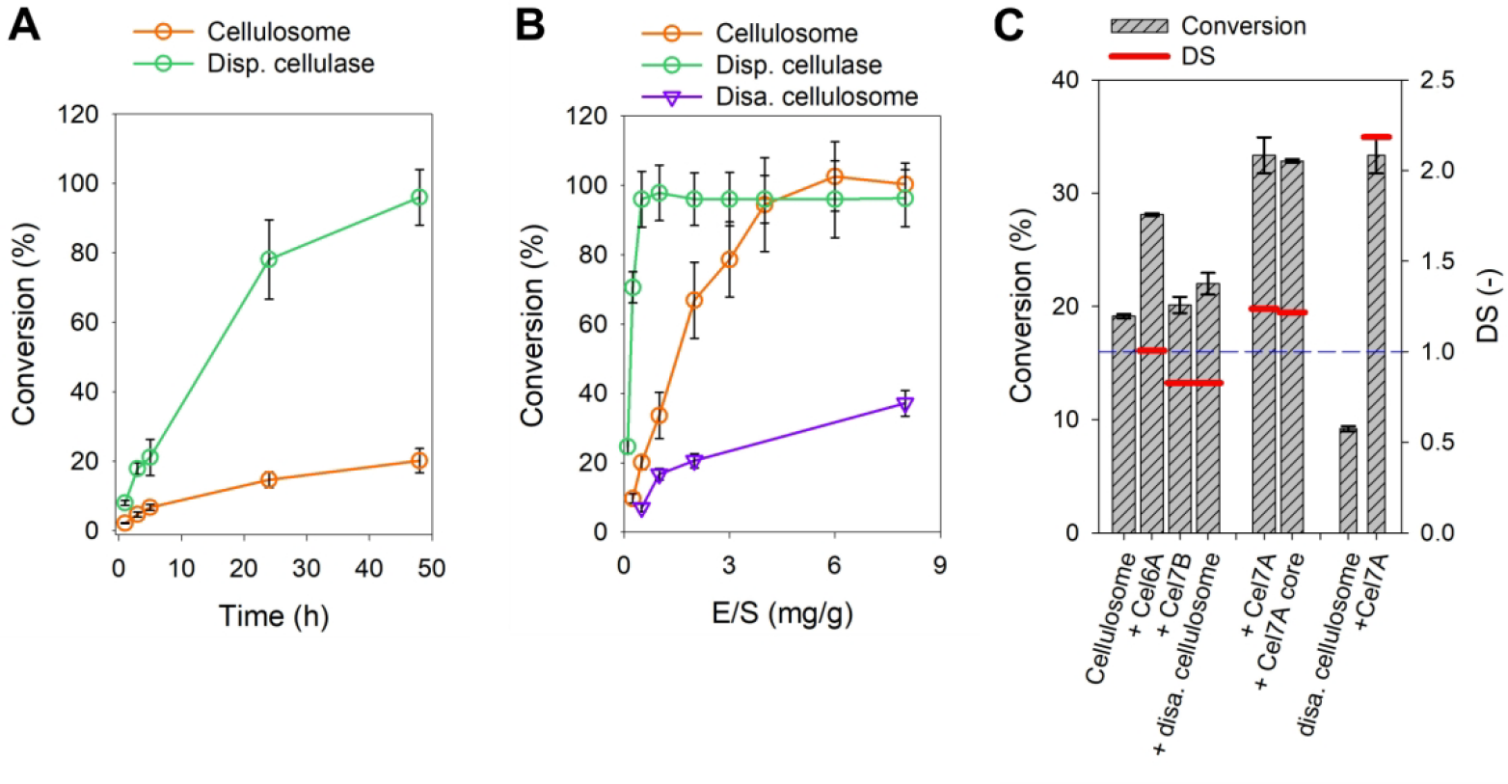
Cellulose hydrolysis by dispersed and complexed cellulases. The conversion was calculated on the basis of the amount of liberated glucose. If not stated otherwise, substrate concentration, temperature, and enzyme loading were 1.0 g/L, 55 °C, and 0.5 mg/g, respectively. (A) Hydrolysis time course of bacterial cellulose using either complexed (cellulosome) or dispersed cellulases. (B) Conversion after 48 h using various loadings of cellulosome, dispersed cellulases, and disassembled cellulosomes. (C) Synergy between the cellulosome and individual dispersed cellulases after 24 h. The cellulosome was present at 1.0 μg/mL and supplemented with either Cel6A (0.5 μg/mL), Cel7B (0.5 μg/mL), disassembled cellulosome (0.4 μg/mL), Cel7A (0.5 μg/mL), or the Cel7A core protein (1.0 μg/mL). The disassembled cellulosome (0.8 μg/mL) was supplemented with Cel7A (0.5 μg/mL).

To what extent is the bulk substrate conversion affected by differences in the enzymatic mode of cellulose deconstruction at nanoscale? To address this important question, we performed hydrolysis studies in suspension, measuring the release of soluble sugars from the same cellulose substrate as used in AFM experiments. As shown in Figure 5A, the *T. reesei* cellulases are considerably more active (∼5-fold) than the native cellulosome as regards the specific hydrolysis rate. The activity of the dispersed cellulosome is further decreased by ∼3-fold compared to the native cellulosome. The maximum conversion of substrate shows strong dependence on the enzyme loading when the native and the dispersed cellulosomes are used. In contrast, it is largely independent of enzyme loading for the *T. reesei* cellulases (Figure 5B).

These results suggest that native and dispersed cellulosomes are both lacking a “factor” present in the *T. reesei* cellulases and important for efficient hydrolysis of the bacterial cellulose. Supplementing each cellulosome preparation with an individual fungal cellulase (Figure 5C), we show that cellobiohydrolase Cel7A enhances the activity strongly (∼1.2-fold the sum of the individual activities), while the endoglucanase Cel7B and the nonreducing chain end-specific cellobiohydrolase Cel6A are not effective. The addition of complete *T. reesei* cellulase yields the highest conversion (not shown) but unlike Cel7A alone, the cellulase does not create synergy (see equation S1 in the Supporting Information) with the cellulosome, in accordance with earlier findings.^81,82^ Interestingly, the enhancement due to Cel7A addition is most pronounced for the disassembled cellulosome (∼2.0-fold). Note the requirement of these supplementation experiments to use the added cellulases under “cellulosome buffer” conditions, not fully optimal for their activity. Notwithstanding this, the shown evidence identifies Cel7A as an effective functional complement of the cellulosome’s activity.

Considering that the cellulosome has its own major cellobiohydrolase (Cel48S)^81,83^ which like Cel7A depolymerizes cellulose chains processively from the reducing end,^10^ we asked what the Cel7A has, and the dispersed Cel48S apparently lacks, that is crucial for synergy with the native cellulosome. Being a strong candidate to make a difference, the cellulose binding module of Cel7A was processed off from the enzyme,^84^ and the isolated catalytic module was examined separately (Figure S13). As shown in Figure 5C, the Cel7A catalytic module is almost identically effective as the intact cellulase, suggesting that synergy with the cellulosome is due to an intrinsic cellobiohydrolase catalytic feature of the Cel7A, independent of interactions from the enzyme’s binding module.

**Figure 6:**
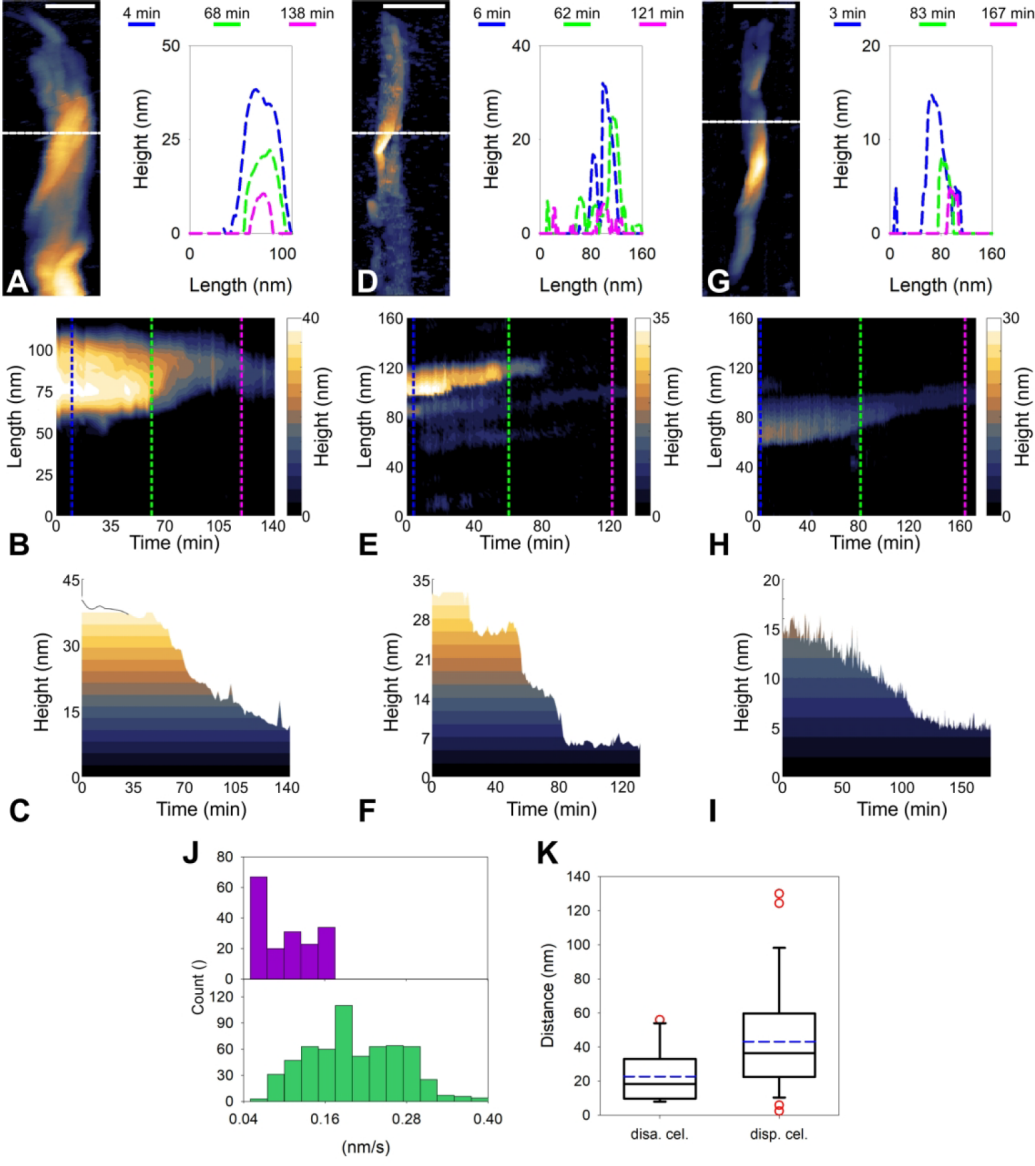
Local degradation efficiency of dispersed and complexed cellulases. Local deconstruction as performed by dispersed cellulases (A, B, C), the cellulosome (D, E, F), and disassembled cellulosomes (G, H, I). (A, D, G) AFM height images were used to select a pixel row (white dashed line) and track the height profiles over time (left). Exemplary height profiles are shown at the beginning, in the middle, and toward the end of the respective degradation process in the corresponding panels (right). (B, E, H) The height progression over time is plotted in a top down perspective allowing a continuous tracking of the time-dependent local changes in fiber height, width, and surface structure. (C, F, I) The maximal height values per pixel row (taken from B, E, H) and time point were used to construct these plots and calculate the mean height per time step size for every pixel row resulted in a distribution of degradation speeds as shown in panel J. (J) Mean degradation velocity measured for dispersed cellulases (green bars) and the disassembled cellulosome (purple bars), respectively. (K) Average processive distance by the disassembled cellulosomes (*N* = 14) and dispersed cellulases (*N* = 30). Median and mean are shown as solid black and dashed blue lines, respectively. Scale bars are 100 nm (A, D, G).

To pinpoint nanoscale effects in substrate deconstruction from which Cel7A synergy with the cellulosome might originate, we analyzed our real-time AFM data for material height reduction at representative fiber cross sections (Figure 6A–C) and so obtained a highly localized assessment of the degradation mode of the respective enzyme system. The height reduction by the cellulases is continuous (i.e., height loss/time step is relatively constant and small compared with the total height loss; Figure 6A–D, G; Figure S14), whereas that by the cellulosome involves discrete steps of much larger size (≥20% of total height reduction; Figure 6B, E, H; Figure S15). The height reduction by the dispersed cellulosome is also continuous (Figure 6C, F, I; Figure S16), but the step size (height/time; median: 0.03 nm/min) is considerably smaller than that of the cellulases (median: 0.19 nm/min) (Figure 6J). The disassembled cellulosome and the dispersed cellulases are highly similar in their basic characteristics of cellulose microfibril deconstruction: both use effectively the same defect regions of the microfibril to initiate their attack and then remove material in the lateral direction (Figures 3, 4; Movies S3, S4 and S5, S6). Considering this, the enhanced degradation speed of the cellulases very plausibly derives from an improved processivity of the enzymes, in particular Cel7A,^85,86^ as compared to the dispersed enzymatic subunits of the cellulosome, in particular the quintessential Cel48S.^83,87^ We therefore analyzed the lateral distance of the individual degradation events in more detail (Figure 6K) and find that indeed, the cellulases, or the Cel7A and Cel7B combination of purified enzymes, involve a much larger “length of action” (∼1.9-fold; median: 34 nm) in each event than the dispersed cellulosome (median: 18 nm).

## Discussion

Evidence from the current study provides a mechanistic correlation between the molecular assembly state of cellulases and the nanoscale characteristics of cellulose deconstruction by the enzymes. From this, a new dynamic interpretation of cellulase synergy obtains and an important relationship with hydrolytic efficiency is suggested. Direct observations were made possible from a dedicated cellulose substrate preparation. Despite its simple macroscopic morphology (a single fiber built from a bundle of microfibrils), this substrate involved the essential elements of supramolecular nanoarchitecture of native cellulose, as pertinent to the analysis of different enzymes working in synergy for deconstruction. In particular, the individual crystalline microfibrils comprised amorphous nanodomains regularly interspersed between domains of high order of the molecular chain organization. The amorphous nanodomains were the preferred common initiation sites for substrate degradation by the cellulases irrespective of their assembly state. Although widely used, the idea of “amorphous material” in enzymatic cellulose deconstruction is vague.^88−90^ Our findings define it, structurally and functionally, in terms of nanoscale defects of cellulose chain organization in the microfibril,^21,22,70^ notably distinct from larger-scale deformations/dislocations of the fiber’s microfibril composite structure.^27,59^ We notice that the results have relevance to inform cellulose pretreatment for facilitated enzymatic degradation.^6,91,92^ Disintegrating the fiber structure solely in terms of organization of the different microfibrils seems not enough for optimum efficiency. The microfibril nanostructure is identified as additional target for the pretreatment. Our argument, of course, recognizes the central task of practical pretreatment, which is to disentangle the stable composite structure of the lignocellulose substrate, with hemicellulose and lignin present in tight association with the cellulose. For the mechanistic inquiry pursued, the results are important in showing that different cellulase systems are comparable on the basis of effectively the same cellulosic nanomaterial degraded.

Stable enzyme complexes direct the catalytic deconstruction transverse to the longitudinal axis of the cellulose fiber. The size of local cuts into the microfibril surface reveal the dynamic reach of action (∼30 nm) for a single cellulosome molecule absorbed in place. The cuts destabilize the fibril bundle architecture proximally, which can occasionally appear as fraying of the fiber’s macrostructure. Others have phenomenologically noted frayed regions in cellulose particles upon treatment with the cellulosome,^81,82^ but the actual deconstruction events leading to it were not resolved. Dispersed enzymes promote substrate deconstruction in the opposite (lateral) direction, observable in multiple events of ablation of surface-accessible single microfibrils. The directionality change (transversal → lateral) reflects the release of restraint on cellulase processivity due to stable complex formation. Directional preference of cellulases (lateral) and cellulosome (transversal) was previously also noted in studies of the degradation of soft wood kraft pulp.^49^ The lateral deconstruction of the microfibrils by the dispersed cellulases involves cellulose chain depolymerization from the reducing end, which is a specificity represented by their Cel7A.^10^ In the dispersed cellulosome, this specificity is represented by Cel48S.^81,93^ Both are major constituents (≥24–40% of total enzyme protein)^10,80,94^ of the respective cellulase system. Although distinct evolutionary and mechanistically,^77^ the Cel7A and Cel48S feature substrate binding pockets of closely related subsite topology: 2 (Cel48S)^95^ or 3 (Cel7A)^96^ glucose units are accommodated after, and 7 glucose units (both enzymes) are accommodated before, the catalytic site of bond fission. Unlike Cel7A,^10,76,85,86,97^ the processivity of Cel48S has not been examined, as far as we know. However, kinetic data imply a processivity on bacterial cellulose considerably higher for Cel7A (∼61,^98^ 88 ± 10^99^) than Cel48A (∼15).^100^ Cel48A is a cellobiohydrolase related to Cel48S by common membership to glycoside hydrolase family GH48 (∼72% sequence identity for the GH48 core). Cel48A is found prominently in dispersed-type cellulases of bacteria.^100,101^ On cellulose pretreated with endoglucanase, the processivity of Cel7A was still ∼34 ± 2.^99^ Since each processive step is ∼1 nm (the size of the cellobiose product released^85^), the AFM-observed lateral distance of ∼34 nm (Figure 6K) for microfibril degradation by the cellulases reflects the processive length of chain depolymerization by Cel7A working in synergy with endoglucanase(s). The shorter lateral distance of ∼18 nm for the dispersed cellulosome may indeed be due to Cel48S being less prominently processive than Cel7A. The Cel48 cellobiohydrolases seem to be only moderately efficient in cellulose hydrolysis, typically considerably less than Cel7A. Of note, the cellobiohydrolase Cel6A shows reduced processivity compared with Cel7A,^102,103^ and it is less permissive than Cel7A for the cellulose substrate used.^104^ The combination of these properties of Cel6A could explain the enzyme’s inability to synergize with the cellulosome. Investigations of *Thermobifida fusca* Cel48A degrading bacterial cellulose showed that the enzyme’s efficiency in hydrolysis was indeed correlated with the processivity.^100,101^ In summary, the picture revealed is that assembly on the scaffold protein restricts the laterally directed activity of processive enzymes in the cellulosome. Dispersion of the enzymes removes the confining force, and so lateral activity becomes dominant in a disassembled preparation of the cellulosome (see ref (74)). Although analogous in the principle, the nanoscale cellulose deconstruction by the dispersed cellulosome falls short in processive efficiency of that of naturally dispersed cellulases, arguably explainable by the properties of the respective reducing chain end-cleaving cellobiohydrolases.

The fundamentally different modes of cellulose deconstruction by cellulases and the cellulosome raise the important question of their relative efficiency in enzymatic hydrolysis of the bacterial cellulose fibers. The cellulosome is just ∼20% as active on the bacterial cellulose as the naturally dispersed cellulases. From our visualization results, the cellulosome is identified as a specialized, fiber-fragmenting nanomachine.^38^ The cellulases in contrast are more globally effective toward full-scale depolymerization of the solid substrate into soluble sugars. This is seen not only in the higher intrinsic hydrolysis rate of the dispersed cellulases but also in the fact that the maximum sugar release is almost independent of the enzyme loading for the cellulases (as expected for an ideal catalytic system), whereas it shows pronounced dependence on the enzyme loading for the cellulosome. Extension of these conclusions to other cellulose substrates should be made with due caution, keeping in mind the purposefully selected, special characteristics of the bacterial cellulose fibers used here. The use of a pure cellulose substrate has eliminated various possible effects of hemicellulose and lignin on the enzyme activity.^5,82^ Additionally, the simple fiber structure of the bacterial cellulose used minimizes the influence of the substrate morphology at fiber aggregate and particle levels. These reservations notwithstanding, we show that the cellobiohydrolase Cel7A (or only its catalytic module) that is enabled to freely disperse makes most of the difference between the two cellulase systems. The Cel7A shows excellent potential to functionally complement the cellulosome’s hydrolytic activity. Its effect is understood as completing the full synergistic cycle between local concentration and dispersion of the individual enzymes during their attack on the solid substrate: the Cel7A adds the dynamic element of dispersion which is low in the cellulosome for the benefit of concentrating the enzymes. Importantly, evidence that other dispersed cellulases such as Cel7B and Cel6A are ineffective in complementing the cellulosome, identifies the Cel7A specifically for this task. The ability of Cel7A to move processively over long distances of cellulose chain seems to be key. Conversely, the interesting problem arises of how dispersed cellulases manage the local concentration of their enzymes for optimum cooperative function. Findings of the current study encourage speculation about dynamic clustering of individual cellulases on the cellulose surface. The cellulase clusters envisioned are transient and disperse under the processive movement of the Cel7A molecules originally engaged in their formation. Importantly, the clusters here considered are productive, and involve enzyme synergy, in substrate deconstruction. They must therefore be distinguished sharply from unproductive “collision clusters” of processive enzymes that have previously been observed.^55^ A recent AFM study of endo- and exochitinases hydrolyzing chitin supports the idea of transient enzyme clusters having a role in solid substrate deconstruction.^9^

Lastly, our results suggest a nuanced view on a general concept of multienzyme complex bioengineering for efficiency-enhanced polymer substrate degradation. The exploitable proximity effects, resulting from locally concentrating enzymes of synergistic function on a tailored scaffold protein, were demonstrated in seminal studies of engineered cellulosome parts (designer cellulosomes).^40^ However, performance characteristics of the native cellulosome shown here reveal limitations on the enhancement of deconstruction efficiency that enzyme assembly into stable complexes could possibly achieve. The fundamental issue is that enzyme complexation is realized by necessity at the cost of enzyme dispersion. A “hybrid approach” seems promising wherein stable enzyme complexes are made to work together with complementarily active enzymes of the dispersed type. The ultimate challenge in multienzyme biocatalyst development for polymer degradation would be protein engineering for coordinated formation and dispersion of transient clusters of synergistic enzymes on the solid substrate.^9^

## Supporting Information Available

Detailed description of the materials and methods involved in the preparation and analysis of bacterial cellulose; sample preparation and parameter selection for AFM observations; AFM data analysis; enzyme production, purification, and characterization; synergy experiments; supporting figures; and supporting movie captions (PDF)
Fragmentation of bacterial cellulose fibers by the cellulosome (Movie S1) (MOV)
Disruption of big fiber part caused by the cellulosome and the AFM tip (Movie S2) (MOV)
Peeling of bacterial cellulose by dispersed cellulases (Movie S3) (MOV)
Thinning of bacterial cellulose by dispersed cellulases (Movie S4) (MOV)
Longitudinal degradation of bacterial cellulose by the disassembled cellulosome (Movie S5) (MOV)
Thinning behavior of the disassembled cellulosome on bacterial cellulose (Movie S6) (MOV)

## Supplementary Material

cs1c03465_si_001.pdf

cs1c03465_si_002.mov

cs1c03465_si_003.mov

cs1c03465_si_004.mov

cs1c03465_si_005.mov

cs1c03465_si_006.mov

cs1c03465_si_007.mov
