# Supplementary material for: Processive Enzymes Kept on a Leash: How Cellulase Activity in Multienzyme Complexes Directs Nanoscale Deconstruction of Cellulose
Source: ACS Catal. 2021 Oct 25;11(21):13530–42. doi: 10.1021/acscatal.1c03465 (PMC8576811; doi:10.1021/acscatal.1c03465)
Supplement: Supplementary file 1 — cs1c03465_si_001.pdf [file cs1c03465_si_001.pdf]

Supporting information

Processive enzymes kept on a leash: how cellulase activity in multienzyme complexes directs nanoscale deconstruction of cellulose

*Krisztina Zajki-Zechmeister[1], Gaurav Singh Kaira[1,2], Manuel Eibinger[1], Klara Seelich[1], Bernd Nidetzky[1,2]**

[1]Institute of Biotechnology and Biochemical Engineering, Graz University of Technology, Petersgasse 10-12/1, 8010 Graz, Austria.
[2]Austrian Centre of Industrial Biotechnology, Petersgasse 14, 8010 Graz, Austria.

Corresponding Author

*Correspondence should be addressed to B.N. (bernd.nidetzky@tugraz.at)

Materials and Methods

Unless stated, all chemicals were of the highest purity available from Carl Roth + Co KG (Karlsruhe, Germany).

Cellulose substrate – Preparation of bacterial cellulose.

Acetobacter xylinum (46602, DSMZ-German Collection of Microorganisms and Cell Cultures GmbH, Braunschweig, Germany) was cultivated in sterile yeast peptone mannitol medium (360, DSMZ) containing 5 g yeast extract, 3 g peptone, 25 g mannitol in 1 L of ultrapure water. Inoculation of 2 mL bacterium in 200 mL medium was done in 500 mL Erlenmeyer flasks. This flask size was chosen to allow easy handling while providing a sufficient gas-liquid interface. Static cultures were incubated at 30 °C for 14 days overall.

During this time, the aerobic bacteria synthesized two cellulose films. After 7 days, the primary formed film was sunk to the bottom by mildly shaking; thus, the interface for formation of the secondary film was made available. The secondary film was formed during the following 7 days. Only the secondary film was harvested for further use, because it showed consistent visual and morphological properties, in contrast to the primary film. It was rinsed with ultrapure water and incubated in 1 M sodium hydroxide overnight, at 4 °C with minimal magnetic stirring to remove biomass. Repeated washing and incubation in ultrapure water was performed to remove cell debris, residual sodium hydroxide and media components until the water bath reached pH 7. The washed films were stored at 4°C in ultrapure water and were therefore never dried.

Cellulose substrate – Isolating fibers

The cellulose film was transferred into a beaker filled with sterile ultrapure water and mechanically disintegrated with a hand blender for 5 min. The viscous cellulose water mixture was diluted at least 1:10 with ultrapure water to prevent hydrogel formation. Further

disruption was done by ultrasonic homogenization with a Sonic Dismembrator (Fisherbrand™ Model 505, Fisher Scientific GmbH, Im Heiligen Feld, Germany) operated with a 6A/4V titanium alloy probe head (Fisherbrand™ FB4406, Fisher Scientific GmbH). The probe head was kept in the middle of the sample to improve liquid circulation. The Dismembrator was used in pulse mode with a pulse time of 2 s, a pause time of 5 s and an intensity of 40 %, while constantly cooling the sample in an ice bath. A maximum sonication time of 20 min was chosen. This ensured that single cellulose fibers were released from the three-dimensional fiber network that the bacterial cellulose film represents (Fig. **S2**) and at the same time, mechanical disruption of the crystalline nanostructure of the cellulose fiber was minimized.

Cellulose substrate - Characterization

The bacterial cellulose was analyzed for its crystallinity according to Segal et al (1). The content of cellulose allomorph Iα was determined according to Wada et al (2).

X-ray diffraction patterns where measured on the Elettra beamline XRD2 (Trieste, Italy), using an energy of 12.398 keV and a Pilatus 2M detector placed in a distance of 134.2 mm from the sample. An approximately 1 mm^2 small piece was cut from the wet cellulose sheet and mounted on a metal loop aligned perpendicular to the beam. Diffraction patterns were acquired for 90 s at room temperature. Note: additional treatment of the sample with a cryo-stream resulted in water crystal spots in the diffraction pattern. The procedure used at room temperature resulted in quick drying of the sample, so a large part of the intensity measurement was contributed from dry cellulose sheet. The measurement was done for two different regions of one cellulose sheet. The empty metal loop was also measured and subtracted from the signal. Integrating the Debye-Scherrer rings resulted in 1D intensity signals, which were linearly baseline corrected and normalized with data analysis software (OriginPro 2019b, OriginLab Corporation, Northhampton, MA, US) (Fig. **S4A**). The peak height calculation after the method of Segal el al (*1*) resulted in crystallinity indices between

85 % and 91 %. This was in accordance with the previously reported high crystallinity of bacterial cellulose (3, 4).

The d spacing calculations and Z-plot generation according to Wada et al (2) showed that all regions from the cellulose sheet were in the Iα rich category. Individual d spacings used to calculate the ratio regions (white dotted lines in Fig. **S4B**) were taken from published data for *Cladophora* algal cellulose (2). Because bacterial cellulose was expected to behave similarly as algal cellulose regarding its allomorph ratios, an estimation of the I_{α}:I_{β} ratio was therefore possible. The cellulose sheet showed variation of the I_{α}:I_{β} ratio between 10:0 and 9:1, depending on the region analyzed.

AFM – Real time experiments

Atomic force microscopy (AFM) measurements were performed with a commercially available device (Dimension Fast Scan Bio™, Bruker, Billerica, MA, USA). The controller (Nanoscope V, Bruker) was operated with the included software (NanoScope 9.2, Bruker). The experiments were done in tapping mode in liquid environment.

Unless stated otherwise, Fast Scan DSS (Bruker AFM Probes, Camarillo, CA, USA) probes were used with nominal frequency, spring constant and tip radius of 110 kHz, 0.25 N/m and 1 nm, respectively. During laser alignment in liquid environment care was taken that the incoming signal at the detector was at least 1 V.

As substrate, a highly ordered pyrolytic graphite (HOPG) crystal with grade I (SPI supplies, West Chester, PA, USA) was used. Cleavage with adhesive tape was immediately followed by immobilization of bacterial cellulose fibers. This was done by incubating the surface with 300 µL heavily diluted (<0.01 g/L) bacterial cellulose suspension for 15 min. Subsequently, the HOPG surface was rinsed with deionized water. Residue water droplets were removed by spraying carbon dioxide on the surface for 2-3 s.

Mounting the substrate on the AFM stage was immediately followed by generating the liquid environment by pipetting 250 µL of the relevant buffer onto the surface and carefully driving

down the scan head until it was completely immersed in the droplet. During long measurements (≥ 3 h), it was necessary to add further liquid due to evaporation. Unless stated, the measurements were performed at 35 °C. The stage was heated with a temperature controller (Bruker) and the added buffers and enzymes were preheated to this temperature prior to injection.

The equilibration times varied between 10 and 60 min. The system was determined as equilibrated once the signal at the photodiode was stable. The surface was scanned for single, isolated and firmly attached cellulose fibers. Once a suitable fiber was found, the tip was stopped (but not withdrawn) followed by careful injection of 10-100 µL enzyme (15 mg/L) solution into the droplet. This was performed with a 100 µL pipette in 10 µL portions.

AFM - Parameter selection

AFM is a very adaptive technique, requiring iterative adjustment of imaging settings based on the feedback from the apparatus, therefore we report our general approach and systematic regarding imaging settings.

For every image the topography, phase and amplitude information were acquired. Two distinct measurement types were performed, dependent on the focus of the measurement being on the cellulose fiber or the enzymes. Scanning with regard to the structural changes of the cellulose fibers was performed by slow scan rates (> 1 frames/min). For imaging the enzymes, time resolution was improved by choosing small imaging regions (< 0.05 µm^2) and increasing the scan rate until the adjustment of feedback parameters no longer resulted in improved image quality. In more detail, tracking errors or other artefacts became predominant, resulting in the enzymes not being recognizable anymore. With this procedure, it was possible to achieve a maximum measurement speed of 1.7 frames/s. In any case, the spatial resolution was adjusted to 2 nm/pixel or better.

Settings regarding the force load on the sample were chosen individually for each measurement. The Drive Amplitude was set to the smallest possible voltage while still being

in a stable measurement region, which was monitored by the phase image. Sudden phase jumps between scan lines or after objects, regardless of Amplitude Setpoint adjustments, were taken as indications for a too small Drive Amplitude. Therefore, the Drive Amplitude was increased in 5 mV steps until the signal stabilized. The Amplitude Setpoint was adjusted to just below contact so as to prevent any disruption of the enzymes or a change/destruction of the cellulose. Amplitude Setpoints of 70-90 % resulted in phase shifts under 20 °, enabling minimally invasive visualization. Generally, a constant Setpoint was used during a measurement sequence, however, in case of any z-drift the Setpoint was adjusted accordingly. The Integral Gain was set just below resonance (and adaptively adjusted to stay there) and the Proportional Gain was kept at a low value (about 3 – 5 times the Integral Gain value). To increase resolution further, the z-range of the scanner was reduced to half of its maximum range.

AFM – Nanomechanical characterization of cellulose fibers

Peakforce QMN was done in liquid at room temperature using a Dimension Fast Scan Bio™. RTESPA-150 (Bruker AFM Probes, Camarillo, CA, USA) probes were used with nominal frequency, spring constant (*k*) and tip radius of 150 kHz, 5 N/m and 8 nm, respectively. Preparation of bacterial cellulose and immobilization on HOPG was done as described above. Prior to each measurement the probes deflection sensitivity was measured on HOPG and *k* was calibrated by thermal tuning method. The effective tip radius was estimated by using the nominal tip radius and opening angle.

Typical scanning size was either 1 × 1 µm², 2 × 2 µm² or 5 × 5 µm² and digital resolution was set to either 256 × 256 pixel or 512 × 512 pixel, respectively.

For imaging, the peak force set point was set to 15 nN and the cantilever was oscillated at 2 kHz. The Scan rate was adapted based on the imaged area to avoid artifact formation but was typically ≤ 1 Hz.

Data evaluation was done using Gwyddion (Version 2.55.

AFM – Image processing and video construction

Processing of the images shown in the time-lapse movies was done by an automated MATLAB routine (developed in R2017b, Version 9.3.0.713579). Unless stated otherwise, data correction was only performed for topography channel data. The routine was inspired by generally used consecutive processing steps in single image processing with established software, like Gwyddion. It consists of three main steps: object masking, data manipulation and scaling.

Masking resulted in a classification of every data point in either object or background. This was done by, on the one hand, finding the edges of the objects and, on the other hand, by finding the surfaces of the objects. The combination of both produced the mask. The edges of the objects were identified by calculating the numerical gradient for every pixel in x- and y-direction. If the root sum square of both gradients exceeded a user defined "gradient parameter" (GP), the pixel was identified as object and masked. The surfaces of the objects were identified by exploiting that the absolute numerical values of the surfaces and background lied in other ranges. The median was calculated for every row and the overall average of all medians was defined as background. If a pixel exceeded the sum of the average median and a user defined "median parameter" (MP), the pixel was masked. Prior to the median process, a rough baseline correction was performed by fitting a plane through all datapoints using least square method. This was merely done to avoid also masking the background in cases of pronounced tilts and should not be confounded with the actual tilt correction performed in the data manipulation part.

GP as well as MP were held constant within the entire measurement sequence. Appropriate choice of the parameters was critical for the quality of the masking process (Fig. **S5**).

The obtained mask was used in all actual data manipulation steps that followed. The tilt of the data was corrected by fitting a plane through all data points marked as background with least square method. The plane was then subtracted from every pixel in the raw image (Fig. **S6**).

Mismatching profiles of pixel rows were corrected by row alignment. By user choice, either a "Degree 0" or "Degree 1" correction was performed for the entire measurement sequence. Degree 0 determined the mean of the background pixels in every row and subtracted this value from all the pixels in this row (Fig. **S7**). This routine was mainly used for data sets with scan areas below 500 nm - 100 nm. Bigger areas often resulted in additional ascending or descending rows. Therefore, "Degree 1" correction was done, by least square fitting a polynomial of degree one through the background data points in every row and successively subtracting these lines from the whole, unmasked row. If rows consisted of 80% or more masked pixels, no correction was performed in either case for these rows.

Uniform scaling was done by defining the lowest pixel value as zero and choosing an individual maximum value in order to enhance visibility of the features of interest. Every image within a sequence was scaled to this maximum value. The final images were exported in its original pixel size and with a bit depth of 24 to portable network graphic (.png) as well as to Gwyddion compatible files, for further analysis.

Unless stated otherwise, the phase and amplitude channel data were only processed regarding the color range. The lowest value was defined as zero and a maximum value was set by the user. Additionally, it was necessary to counteract two often-occurring problems in the amplitude channel data. Due to feedback errors some pixels can contain outlier values and the histogram of the whole image can be asymmetric. Both cause a shift in the false color scale in the upper color region, resulting in unclear visibility of the region of interest. This was handled by eliciting the number of bins between the peak of the histogram and it's maximum. This number was used to define a new minimum for the histogram (Fig. **S8**). This mirroring of bin numbers resulted in cutting off distorting pixel points and increased visibility of the presented features.

A drift correction routine was adapted from Sugar et al(5). Briefly, a reference image was chosen and compared to the remaining images in the sequence. To determine the number of

pixels an image must be shifted in x-, and y-direction the cross-correlation between both images was calculated. In more detail, utilizing the convolution theorem, the two-dimensional Fourier transform of the reference image was multiplied with the complex conjugate of the two-dimensional Fourier transform of the sequence image. The inverse Fourier transform of this results in a two-dimensional matrix containing the cross-correlation function. The position of the maximum value gives the pixel-values by which the image has to be shifted in both directions to maximize the match between reference and sequence image.

Movies were constructed by using Davinci Resolve (Version 16.2.7, Blackmagic Design) and any highlights included in the movies were implemented with MATLAB.

AFM – Image analysis

Qualitative analysis was done visually by comparing consecutive pre-processed images of measurement sequences. To identify cellulose fibers, additionally to the topography, also the phase channel was considered. The high phase contrast, due to different material properties of the fiber and the HOPG background (6), facilitates improved distinction of the fiber from the background. Detection of enzymes was mainly done by additionally evaluating the amplitude signal. The high edge sensitivity of this signal reacts to the steeper and rougher sides of the enzymes compared to the cellulose fiber underneath, resulting in better detectability. Therefore, it complemented the phase contrast and led to clearer identification of enzymes on the cellulose.

Quantitative analysis regarding the global volumetric degradation and was done in MATLAB. The sum of all pixel entries for each frame was calculated, whereby the sum of the first frame was defined as 100 %. Shortly increasing heights, caused by temporally appearing enzymes where excluded. The temporal evolvement of this percentage is representative of the global degradation behaviour of the enzymes (Fig. **2E** and **3D**).

Quantiative analysis regarding the local mode of degradation was also done in MATLAB. The temporal change of the height profile along the vertical axis of the fiberbundle (Fig. **S9A**)

was tracked. To minimize noise, three neighbouring pixel rows were used for profile generation (Fig. **S9B**). The time dependent height decrease can be represented by plots, where the abscissa represents time, the ordinate the fiberbundle cross-section at a certain position and the color map the height. (Fig. **S9C**), using 15 colors from the color scale. The top view of this plot enables close tracking of the surface and dimensional change of the fiber over time (Fig. **S9D**). The temporal change of the maximum value of every pixel column was tracked and the first derivative was used to calculate the degradation speed. To minimize noise and to disregard suddenly increasing heights, caused by temporally occurring enzymes, the lines where smoothed as follows: Outliers, values that are more than three scaled median absolute deviations from the median, were replaced by the previous non-outlier value. Thereafter, the data was flattened by calculating the moving average of the values, using Gaussian weighing function and a fixed window length. The mean height difference per time step for the moving average was calculated for each individual column (Fig. **S9E**), resulting in the global estimates of degradation speed for the whole fiber (Fig. **6J**). Height increasing steps were excluded to avoid distortion of the mean degradation steps by occasionally appearing enzymes.

Quantitative analysis regarding properties such as fiber length were performed using common tools in Gwyddion (Version 2.55) (7).

Enzyme preparation – Cellulosome

Clostridium thermocellum ATTC 27405 (8, 9) was grown anaerobically in GS-2 media on microcrystalline cellulose as reported previously (10) with some minor adaptations: The cultivation was done in 50 mL at 55 °C with shaking (100 rpm) in 100 mL sealed bottles. The concentration of the sole carbon source (Avicel PH-101) was 10 g/L.

Prior to autoclaving and inoculation, the media were sparged with nitrogen until the redox indicator (resazurin) turned colorless, indicating the absence of oxygen.

Cultivations were done for about 72 h after which most of the Avicel PH-101 substrate had been dissolved.

Solids were removed by 2 times centrifugation (5000 rpm, 20 min) and the cleared supernatant was filtered through a 0.2 µm disposable syringe filter (Minisart, Sartorius, Göttingen, Germany). The buffer was exchanged to 30 mM MOPS, pH 7.0, containing 100 mM NaCl and 10 mM $CaCl_2$, using disposable centrifugal concentrators (Vivaspin Turbo 15, Sartorius) with a 300 kDa molecular mass cut-off. In addition, the sample was concentrated about 10-fold in the process. Purification of the cellulosome was done via size exclusion chromatography using a prep grade column (HiLoad® 16/60 Superdex 200, GE Healthcare, Little Chalfont St. Giles, UK) as reported elsewhere(10).

Fractions containing the cellulosomes were pooled, concentrated (~ 200 µg/mL) and stored at 4 °C. Analysis by SDS PAGE (Fig. **S12A**; see (10)) confirmed that the cellulosome preparation used had the protein composition as expected from the literature (8, 11).

Enzyme preparation – Non-complexed (dispersed) cellulosome

The dispersed cellulosome was prepared according to an earlier method (12), with minor modifications. The method involves disassembly of the enzymatic subunits while the cellulosome is adsorbed on cellulose(12). Therefore, for cellulose binding, 750 µg/mL purified cellulosome was incubated with 150 mg/mL Avicel at 22 °C, 800 rpm for 1 h in a heating block (Thermomixer comfort, Eppendorf AG, Hamburg, Germany). Thereafter, the reaction was centrifuged and the cellulose pellet was washed thrice using 30 mM MOPS, pH 7.0. For disassembling, the cellulose pellet was resuspended in 15 mM EDTA, 30 mM MOPS, pH 7.0, and incubated at 70 °C for 10 min without agitation. The supernatant from centrifugation was collected. The disassembly step was repeated twice and all the supernatant fractions were pooled, concentrated and buffer exchanged using 10 kDa Vivaspin. This preparation was stored in 30 mM MOPS, pH 7.0, and is referred to as dispersed cellulosome.

Analysis by SDS PAGE revealed the presence of the major enzymes of the cellulosome while the scaffoldin CipA was characteristically lacking (Fig. **S12A**) (11).

Enzyme preparation – Dispersed cellulases

Complete (free) cellulase was obtained from the supernatant of the fungus *Trichoderma reesei* (Strain SVG17, Institute of Biotechnology and Biochemical Engineering, University of Technology, Graz, Austria). The SVG17 strain was reported earlier (13) and is an efficient cellulase-producing "wild-type" strain of *T. reesei*. For cellulase production, the strain was grown on wheat straw as described recently(14).

The culture supernatant was processed via centrifugation (Sorvall RC-5Bm, GMI, Ramsey, MN, US) with 4420 g at 4 °C for 20 min and filtered through a glass microfiber centrifuge filter (VectaSpin, Whatman, Maidstone, UK). The clarified cellulase preparation was stored at a final concentration of about 10 g/L at 4 °C supplemented with 0.05 % w/v sodium azide. Analysis by SDS PAGE (data not shown) confirmed that the free cellulase preparation used had the protein composition as expected from the literature (8).

*Tr*Cel7A (the major component of *T. reesei* SVG17 cellulases) was purified from the preparation of the complete cellulase as described recently (15). In brief, cellulase was buffer exchanged to 20 mM triethanolamine, pH 7.0, using disposable centrifugal concentrators (Vivaspin Turbo 15; Sartorius) with a 10 kDa molecular mass cut-off and loaded onto a 6 mL pre-packed column (Resource Q, GE Healthcare) equilibrated with the same buffer. Elution was performed at room temperature with a linear gradient of 0–300 mM sodium chloride over 10 column volumes. *Tr*Cel7A elutes in a discrete protein peak at about 180 mM sodium chloride, clearly separated from other major cellulases such as *Tr*Cel7B and *Tr*Cel6A that elute both at about 0 mM sodium chloride. The purified *Tr*Cel7A was buffer exchanged to 50 mM sodium acetate buffer, pH 5.0, and stored at 4 °C with a final concentration of about 5 mg/mL. Analysis by SDS PAGE (data not shown) revealed a single protein band migrating to a mass of 65 kDa, consistent with the molecular size of the full-length enzyme.

The major endoglucanase Cel7B was obtained commercially (Megazyme, Dublin, Ireland). It is a preparation from *Trichoderma longibrachiatum,* an anamorph of *T. reesei*. Note that the enzyme is nearly identical to its counterpart in *T. reesei* on an amino acid level (identity: 95 %, similarity: > 95 %). The enzyme was stored as provided by the manufacturer at 4 °C. Prior to experiments the Cel7B was buffer exchanged to 50 mM sodium acetate buffer, pH 5.0, using disposable centrifugal concentrators (Vivaspin Turbo 15; Sartorius) with a 100 kDa molecular mass cut-off.

β-Glucosidase from *Aspergillus niger* was from also obtained commercially (Megazyme) and stored at 4 °C as provided by the manufacturer.

Protein concentration of preparations of the complete cellulase and the cellulosome were measured using a commercial kit (Pierce BCA Protein Assay Kit, Thermo scientific, Waltham, MA, US). Concentrations of purified enzyme preparations were determined via UV absorbance at 280 nm using their molar extinction coefficients of 86.760 M^{-1} cm^{-1} (*Tr*Cel7A) and 74.940 M^{-1} cm^{-1} (Cel7B) calculated from their protein sequence using Protparam (Expasy).

Enzyme preparation – *Tr*Cel7A core

*Tr*Cel7A core was prepared using limited papain treatment as described previously (16). Papain was activated in 50 mM ammonium acetate buffer, pH 6.0, at 30 °C and 250 rpm for 30 min. For limited digestion, 200 µg activated papain was added to 1.0 mg purified *Tr*Cel7A and incubated at 30 °C and 250 rpm for 2 h. Thereafter, the digested reaction mixture was immediately buffer exchanged to 10 mM TEA-HCl buffer, pH 7.6, using disposable centrifugal concentrators (Vivaspin Turbo 10; Sartorius) and *Tr*Cel7A core was purified using anion exchange chromatography (Resource Q GE Healthcare). *Tr*Cel7A core purification protocol was similar to *Tr*Cel7A purification as described above. Purified *Tr*Cel7A core was also analyzed on 12 % SDS PAGE (Fig. **S12B**). After purification CBH I core was buffer exchanged to 50 mM sodium acetate buffer, pH 5.0 and stored at 4 °C till further use.

Enzyme characterization – Hydrolysis

All reactions were conducted in triplicates in 1 mL total reaction volume in micro tubes (1.5 mL, Eppendorf AG, Hamburg, Germany). Reactions employing free cellulases were done in 50 mM sodium acetate buffer, pH 5.0, and reactions employing cellulosomes were done in freshly prepared 30 mM sodium acetate buffer, pH 5.5, supplemented with 100 mM NaCl, 10 mM $CaCl_2$, 10 mM cysteine and 2 mM EDTA. Samples were shaken at 500 rpm in a heating block (Thermomixer comfort, Eppendorf AG, Hamburg, Germany) at 55 °C (cellulosomes) or 50 °C (free cellulases). The concentration of bacterial cellulose was 1.0 g/L and β-glucosidase was added to a concentration of 2 μg/mL to all reactions. Reactions were started with the addition of cellulolytic enzyme to a final substrate loading of 2 µg/mg. Samples of the well-mixed suspension were taken at defined time points. In brief, 75 µL were withdrawn and the reaction was stopped by adding 7.5 µL of 1 M NaOH. Subsequently, samples were centrifuged and the cleared supernatant was assayed for glucose using a commercially available kit (Fluitest GLU HK, Analyticon Biotechnologies AG, Lichtenfels, Germany). Specific activities after 1 h were determined to be 9.3 ± 1.1 U/mg for free cellulases and 2.0 ± 0.2 U/mg for cellulosomes. Specific activities for individual cellulases after 2 h were determined to be about 1.2 U/mg and about 0.8 U/mg for *Tr*Cel7A and *Tl*Cel7B, respectively. All values are in agreement with earlier published results using bacterial cellulose (free cellulases, *Tr*Cel7A and *Tl*Cel7B (17, 18)) or microcrystalline cellulose (cellulosome (19)).

Enzyme characterization – Total bacterial cellulose concentration

Bacterial cellulose concentration was determined using the total cellulose solubilization method (20) as reported previously. Briefly, 10 mg bacterial cellulose was hydrolyzed using 72% sulfuric acid for 1 h at room temperature in tightly capped glass tubes. The slurry was mixed at 15 min interval. The hydrolyzed cellulose mixture was diluted to 4 % sulfuric acid and autoclaved at 121 °C for 1 h. Glucose standard was also prepared the same way. Samples

were cooled (overnight at room temperature) and the pH adjusted to 5.0-5.5. Samples were assayed for glucose using a commercially available kit as stated above. The total mass of bacterial cellulose was calculated as anhydroglucose.

Enzyme characterization – Supplementation of the cellulosome in hydrolysis

Hydrolysis experiments were done as described above for reactions of the cellulosome. A standard cellulosome loading of 1.0 µg/mL was used. The cellulosome was supplemented with Cel7A (0.5 µg/mL), Cel7A core (1.0 µg/mL), Cel6A (0.5 µg/mL), Cel7B (0.5 µg/mL) and the disassembled cellulosome (0.4 µg/mL). The Cel7A core loading was selected based on carful comparison with native Cel7A (Fig. **S13**) to guarantee comparable conversions in the supplemented reactions. The disassembled cellulosome was also used as the main enzyme in a loading of 0.8 µg/mL. This was supplemented with Cel7A at a loading of 0.5 µg/mL. The degree of synergy (DS) was calculated for every combination at 24 h by the ratio of glucose produced by the mixture of main and supplemented enzymes (G_{M+S}) to glucose produced by the sum of the individual enzymes ($G_M + G_S$) according to equation S1.

$$DS = \frac{G_{M+S}}{G_M + G_S} \qquad (S1)$$

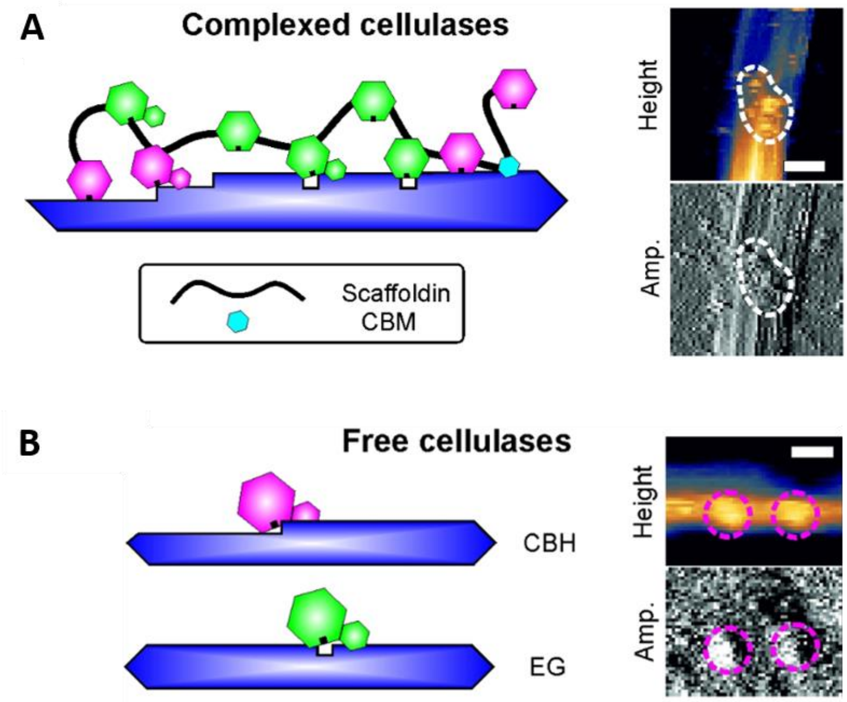

Fig. S1. Schematic representation of the two major cellulase systems. For reason of simplicity, only the major types of cellulase (chain end-cleaving cellobiohydrolase, CBH; internally chain end-cleaving endoglucanase, EG) involved in cellulose degradation are shown. (**A**) The cellulosome featuring assembly of individual CBH (magenta) and EG (green) subunits on a scaffold protein that is equipped with a cellulose binding module (CBM). A single cellulosome from *Clostridium thermocellum* is shown on a cellulose fiber (white circle). Height and amplitude (Amp) images are shown. (**B**) Cellulases comprised of CBH (magenta) and EG (green) that act as ensemble of free enzymes. Each enzyme has a catalytic module linked to a smaller CBM. Two single CBH enzymes from *Trichoderma reesei* Cel7A are shown on a cellulose fiber (magenta circles). Scale bars are 25 nm.

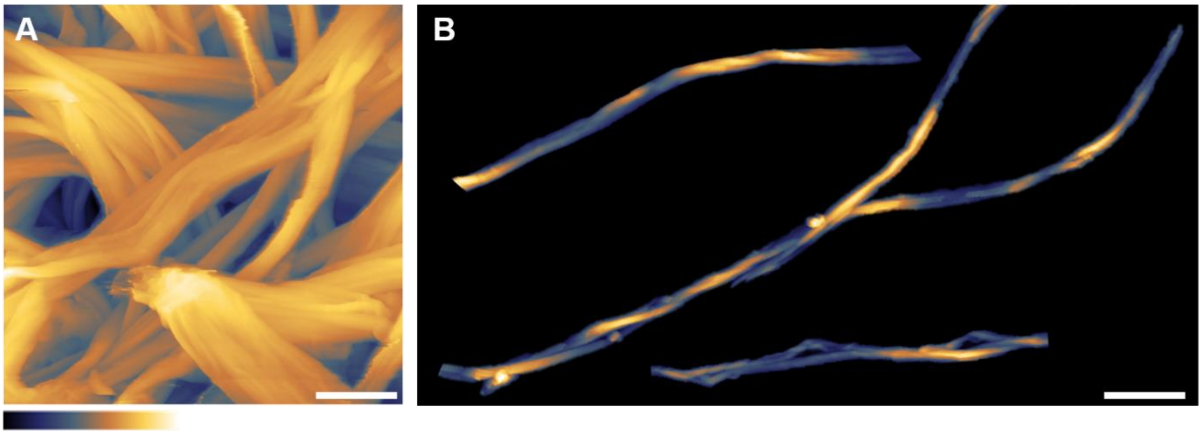

Fig. S2. Conceptual depiction of the sonication process to release single fibers of bacterial cellulose for analysis. (**A**) Cellulose sheet, dried overnight to immobilize it to the HOPG surface, showing the entanglement of the cellulose fibers produced by *A. xylinum*. (**B**) Never dried individual cellulose fibers after 20 min sonication. Both images were measured in liquid AFM. The false color scale used for both panels is shown in panel A. Height ranges are 270 nm (panel **A**) and 35 nm (panel **B**). Scale bars are 200 nm.

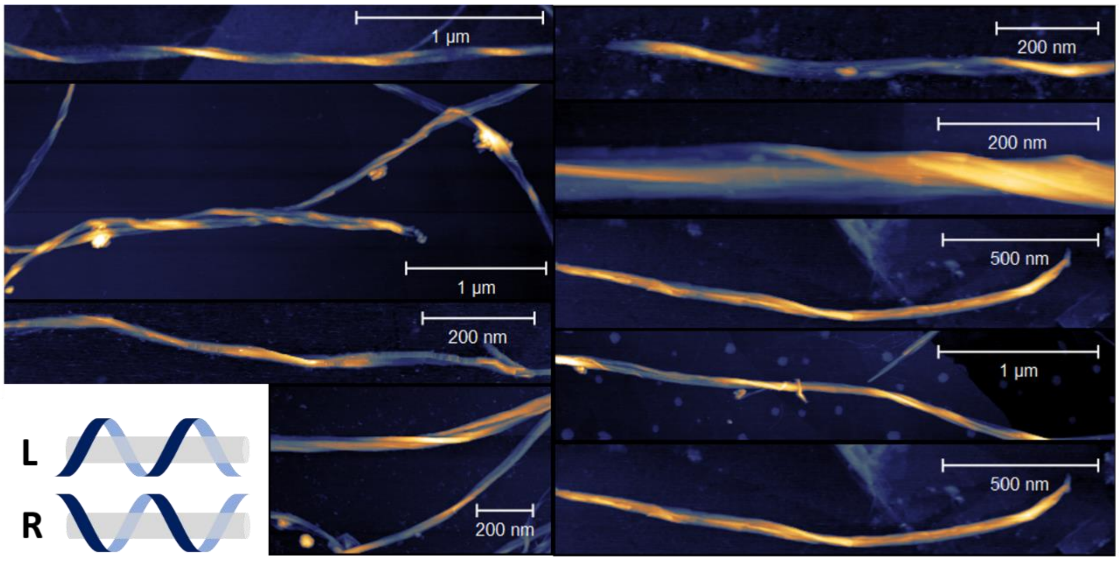

Fig. S3. Analysis of the twist of the bacterial cellulose fibers. Compilation of exemplary fibers with visible right-handedness and a visual aid for the left (L) and right (R) handed route of the twisting. The chirality is an absolute characteristic and is therefore not dependent on the fiber orientation (no turning in any direction can make a right-handed fiber appear like a left handed one). To determine the handedness, one clearly visible twist is necessary. Analysis of fibers meeting this requirement ($\boldsymbol{n} = \mathbf{24}$), resulted in 92 % of them being right handed ($\boldsymbol{n} = \mathbf{22}$).

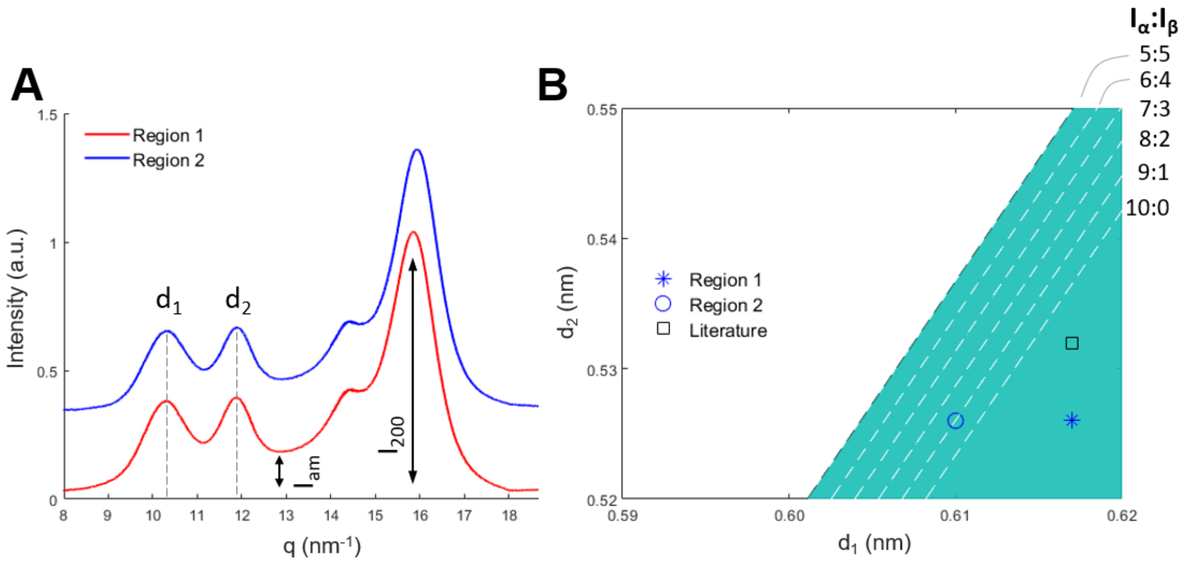

Fig. S4. Results of the XRD analysis of two different regions of the same cellulose sheet from *A. xylinum*. (**A**) 1D intensity signal of both regions (blue and red). (**B**) Z-Plot with highlighted I_α:I_β ratio regions. It can be seen, that both regions (blue ∗ and blue ∘) exhibit high portions of I_α crystalline structure.

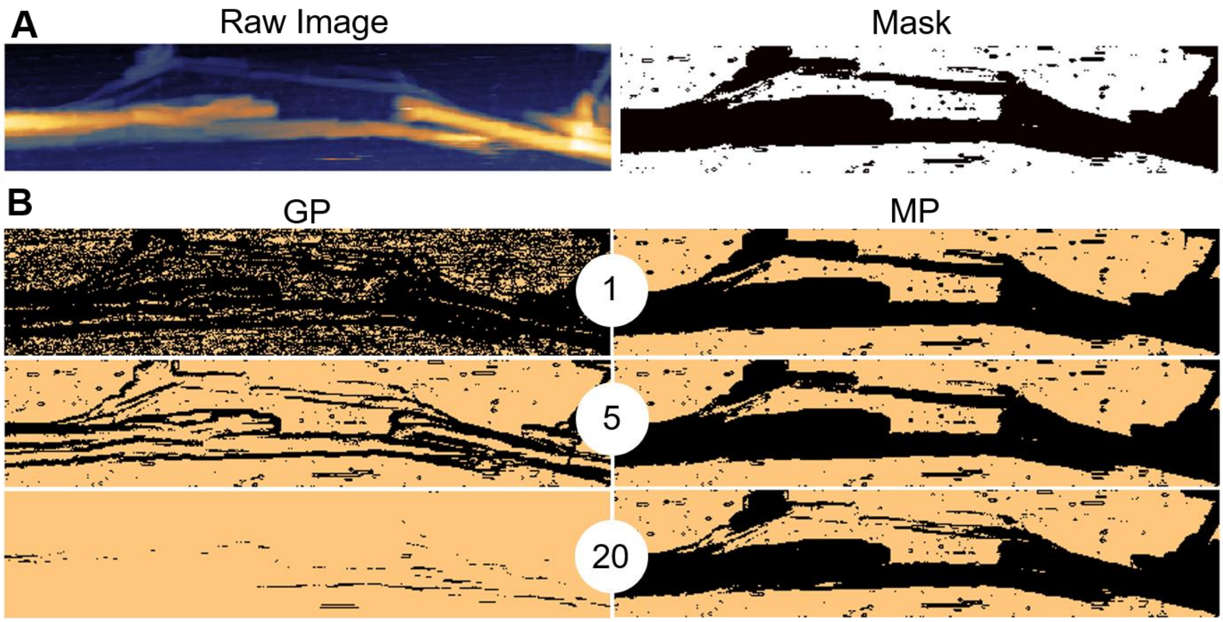

Fig. S5. Illustration of the influence of the user chosen parameters. (**A**) Side by side comparison between raw data and the created mask. (**B**) Influence of different GP (gradient parameter) and MP (median parameter) values. Too small (GP, MP = 1) chosen parameters result in additionally masking of the background, too large parameters (GP, MP = 20) do not mask the edges and surfaces entirely. Appropriately chosen values (GP, MP =5) mask the object accurately.

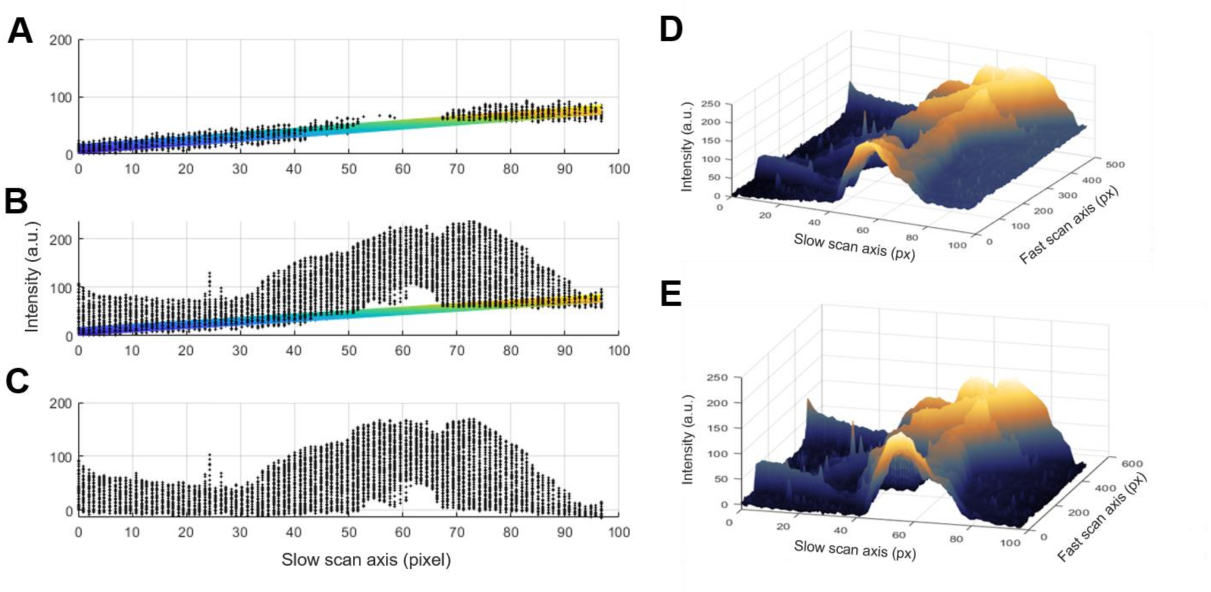

Fig. S6. Illustration of the background subtraction process. (**A**) Scatter plot of background pixels including the fitted plane. (**B**) Scatter plot of all data points previous to background subtraction with included plane. (**C**) Scatter plot of data points after subtraction of the plane. (**D**) 3D representation of the data before subtraction. (**E**) 3D representation of the data after the subtraction step. Especially in the front right part of the image, the improvement can be seen.

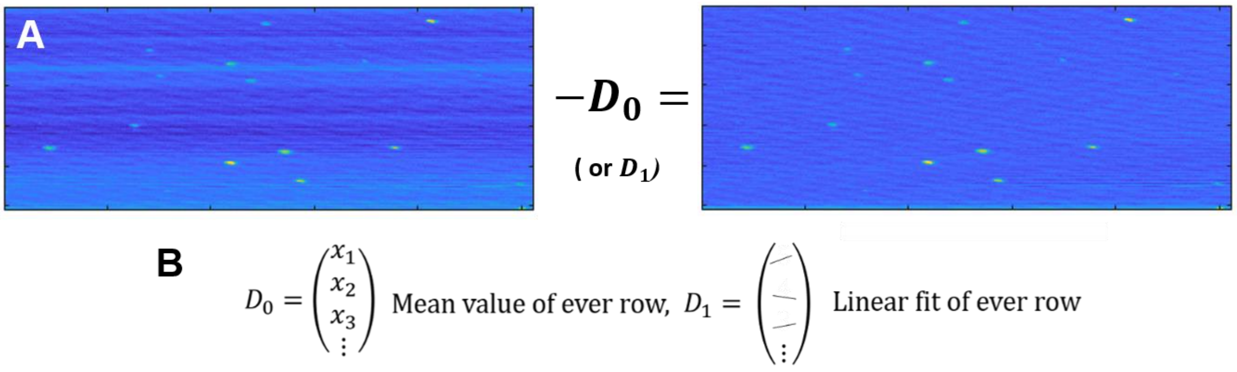

Fig. S7. Schematic of the row alignment. (**A**) Substracting the mean value (or the line) of every row. (**B**) Representation of the subtrahends, containing the mean of each row for degree 0 and the linear equation of the fitted line for degree 1. It can be seen, that after this correction step, most of the line errors are leveld out.

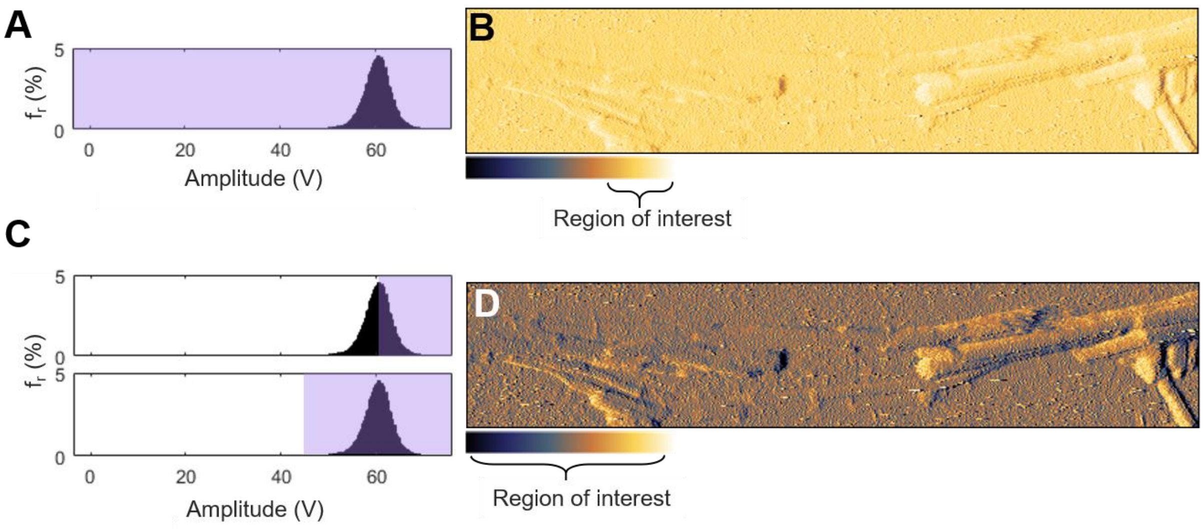

Fig. S8. Illustration of the false color scale correction performed on amplitude images. (**A**) Histogram of the relative frequency (f_r) of all amplitude values in the amplitude image. (**B**) Corresponding AFM amplitude image to the histogram: Although, most data points lie around the 60 V mark, some outliers, which are at the 0 V range, spread the false color scale so that the region of interest is only contained in the upper range of the scale. (**C**) Histogram of the f_r of amplitude values between the peak and maximum value (top) and mirroring the range to the left side (bottom). (**D**) Corresponding AFM amplitude image to the mirrored histogram. The total color range can be used to display the region of interest. The false color scales are 0-72 V (b) and 48-72 V (d).

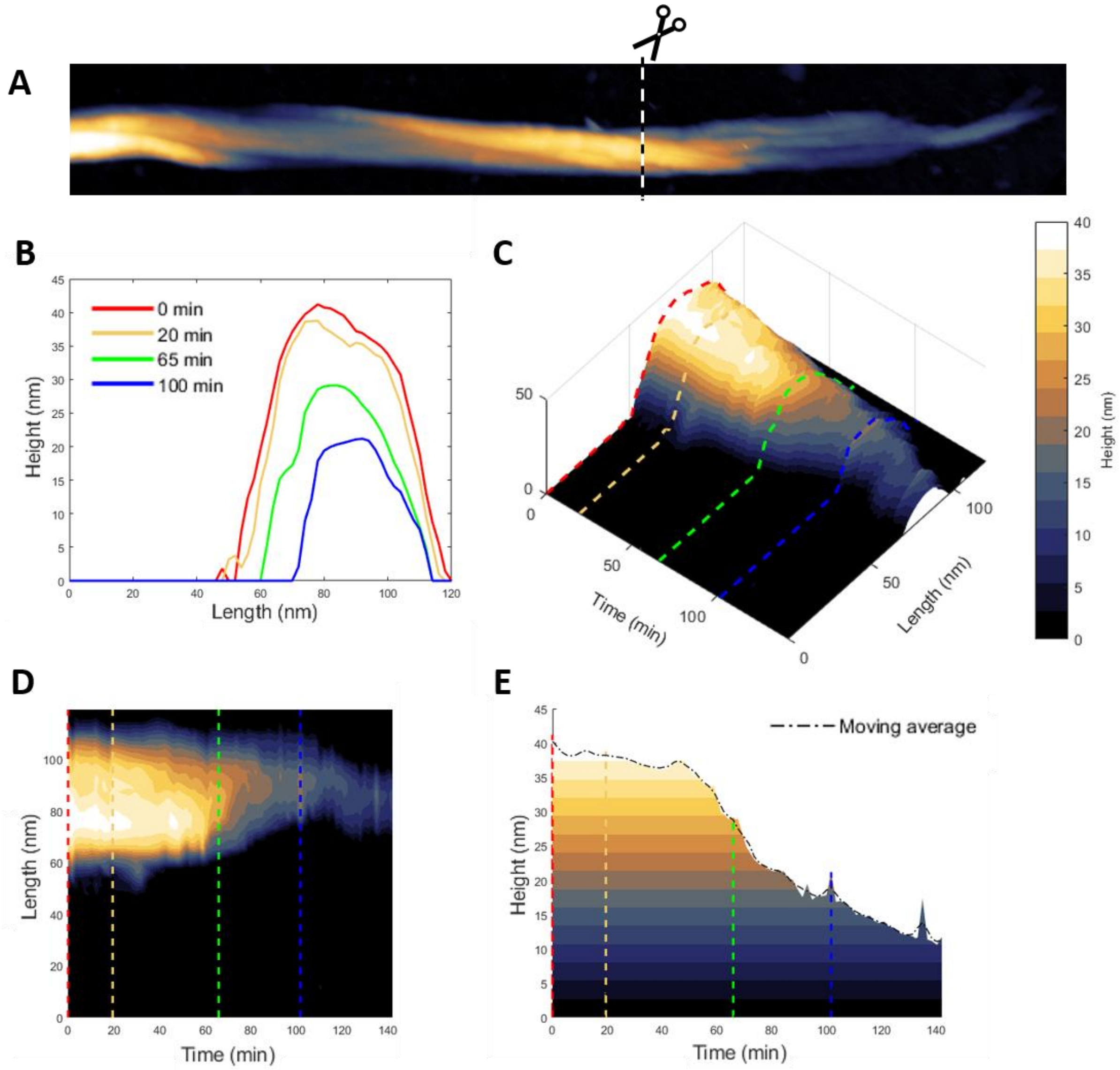

Fig. S9. Illustration of the computation of local deconstruction modes and resulting deconstruction speeds. (**A**) AFM image of the starting frame of a deconstruction experiment with indicated exemplary investigated pixel column. For the actual evaluation every column of the frame was used. (**B**) Exemplary height profiles for the indicated column at different time points in the experiments. It can be seen, that the fiber bundle starts at a maximal height of 40 nm (red) and decreases to half its height after 100 min (blue). Also the width of the fiber bundle shrinks notably. (**C**) 2D plot of all time points for the indicated pixel column with highlighted time points from B, 0 min (red), 20 min (yellow), 65 min (orange) and 100 min (blue) for orientation. The false color scale corresponds to the height. (**D**) Top down perspective of the plot shown in C, again with highlighted time points for orientation. This plot allows the

continuous tracking of the time dependent local changes in fiber height and surface structure. (**E**) Maximum values from every time point, with highlights for orientation. The moving average of this data was used to calculate the mean height decrease per step, resulting in an estimate of the degradation speed for this column.

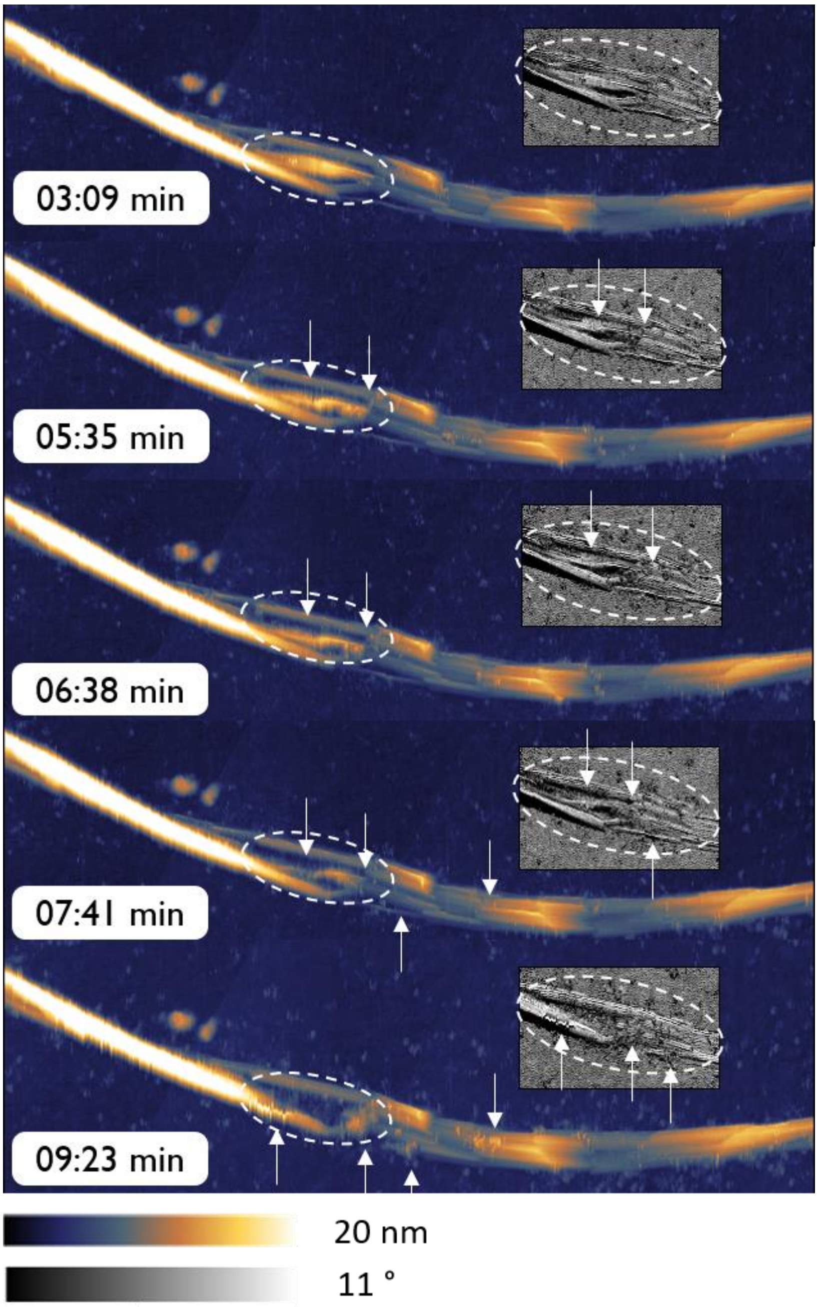

Fig. S10. Detailed view on cellulose degradation by complexed cellulases. The inset shows the material property sensitive phase information revealing structural defects. White arrows indicate defects on the cellulosic surface which are targeted by the cellulosome. The image sequence is taken from Movie S2.

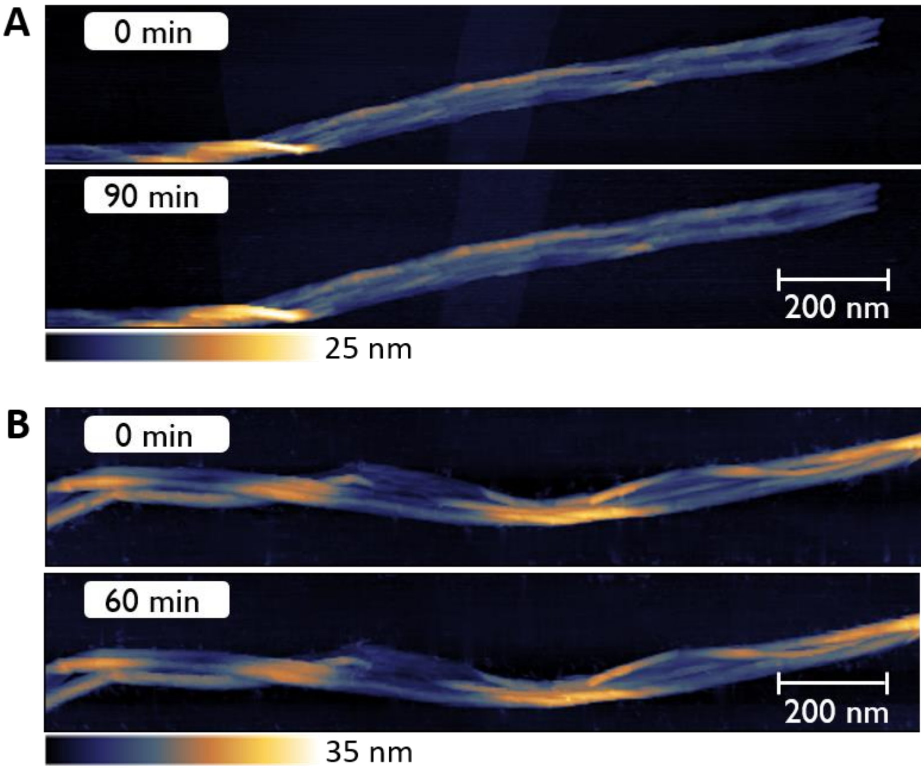

Fig. S11. Stability of cellulose fibers during AFM measurement.

(**A**) The fiber was measured continuously with 4-5 frames per minute for 90 minutes and it can be seen, that without enzymes acting on the fiber, neither structural changes nor tip induced deformation is observed. (B) Another example of a fiber staying unchanged throughout the AFM. It was measured continuously with 3-4 frames per minute for 60 minutes and does not display any destabilization.

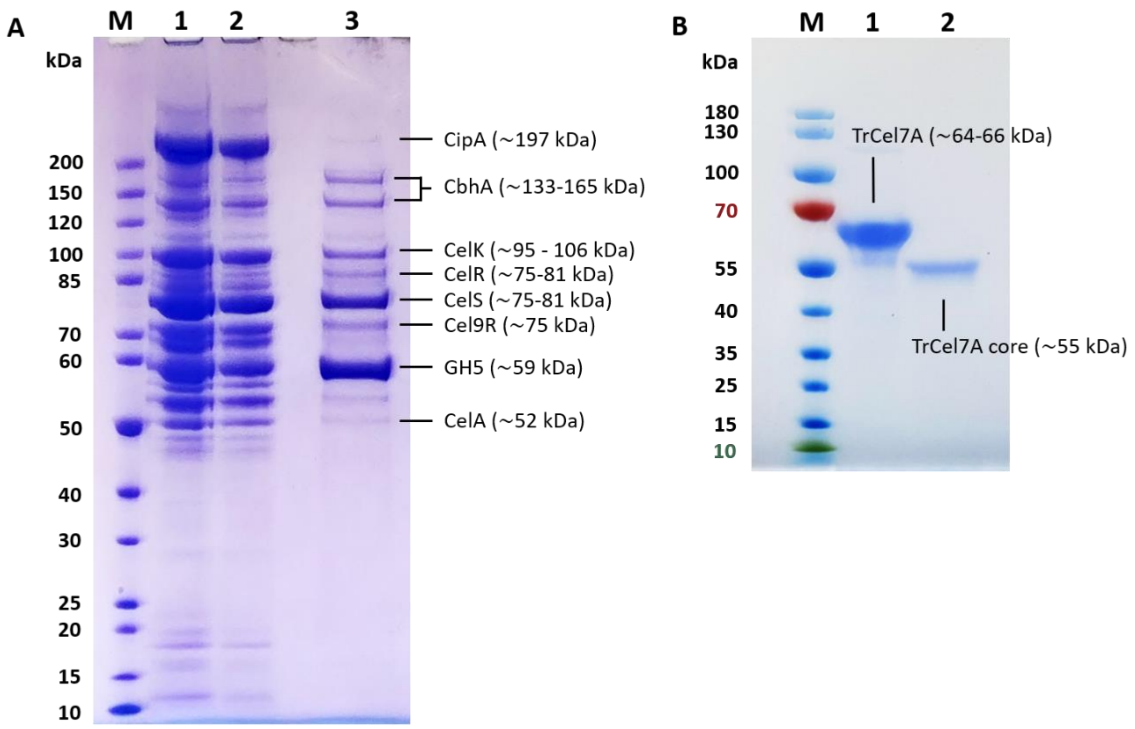

Fig. S12. SDS-PAGE analysis of disassembled cellulosome and *Tr*Cel7A core. (**A**) Disassembly of purified cellulosme obtained from *C. thermocellum*. Next to the marker (M), two lanes of intact cellulosome can be seen (lane 1 and 2). The dispersed cellulosome (lane 3) shows a clear lack of the scaffolding protein CipA. Potential candidate proteins, which were also found abundantly in cellulosomes obtained from avicel grown cells, appeared as prominent gel bands were tentatively assigned based on their molecular weight. (**B**). Analysis of purified *Tr*Cel7A and *Tr*Cel7A catalytic core. Next to the marker (M), it can be seen, that the intact *Tr*Cel7A (lane 1) exhibits a ~9 kDa higher molecular mass than the purified *Tr*Cel7A core (lane 2). This indicates the missing CBM after limited papain digestion.

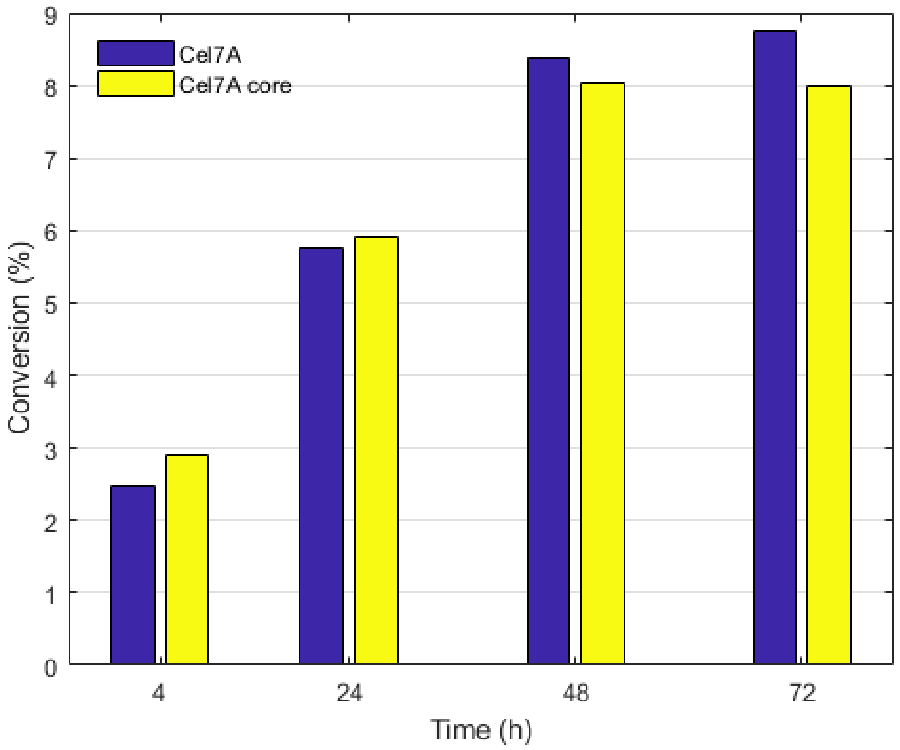

Fig. S13. Comparative bacterial cellulose conversion by Cel7A and Cel7A core. Cel7A needed a protein loading of 0.5 µg/mL to reach the same conversion of substrate as 1.0 µg/mL of the Cel7A catalytic core.

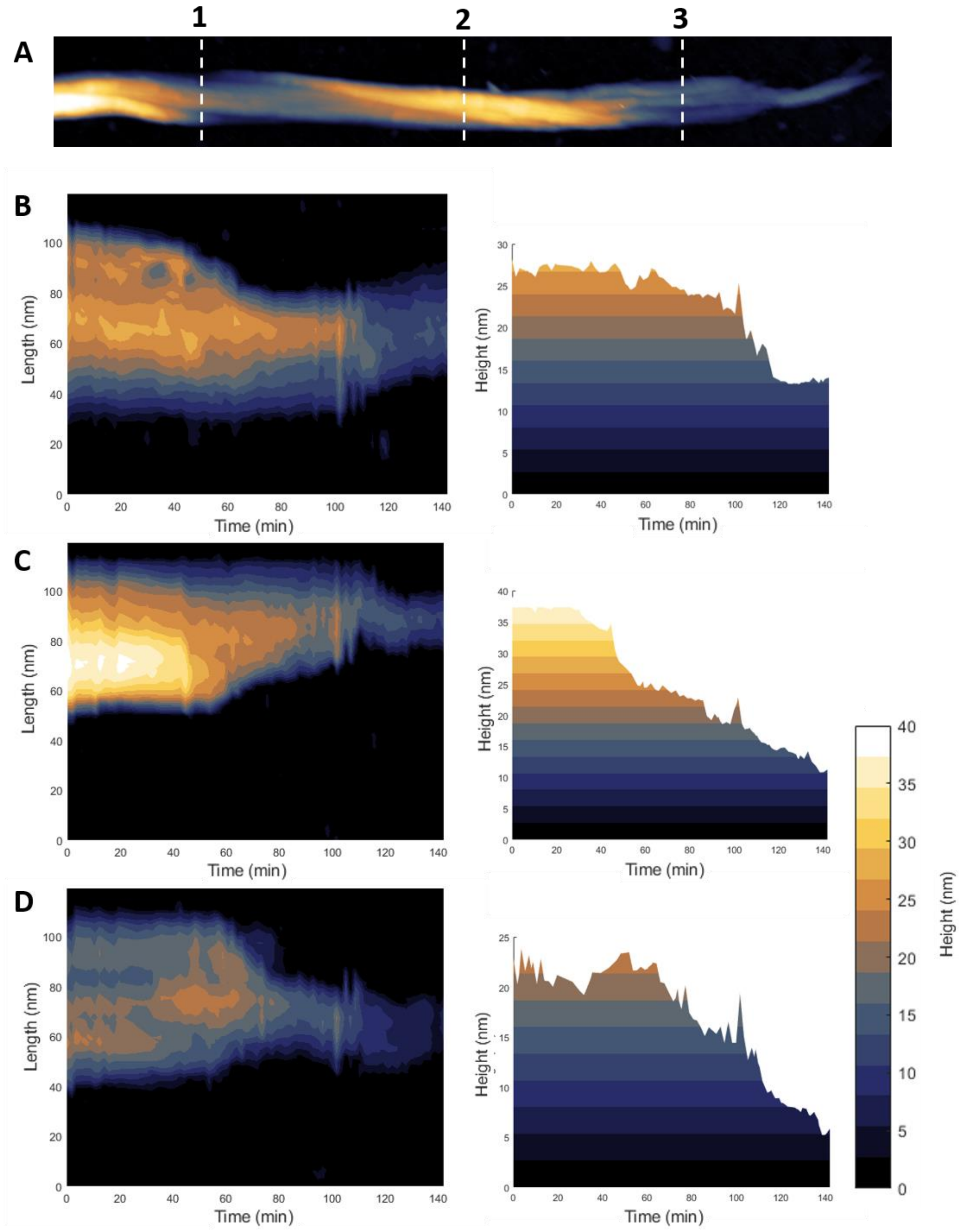

Fig. S14. Additional fiber cross sections showing the same degradation behavior at different positions employed by the dispersed cellulases. (**A**) AFM image of starting frame of the cellulose degradation experiment with indicated exemplary investigated cross sections.

2D plot (left) and maximum values plot (right) of section 1 (**B**), 2(**C**) and 3(**D**). All sections across the fiber bundle exhibit similar continuous degradations styles as depicted in Fig. **6D**.

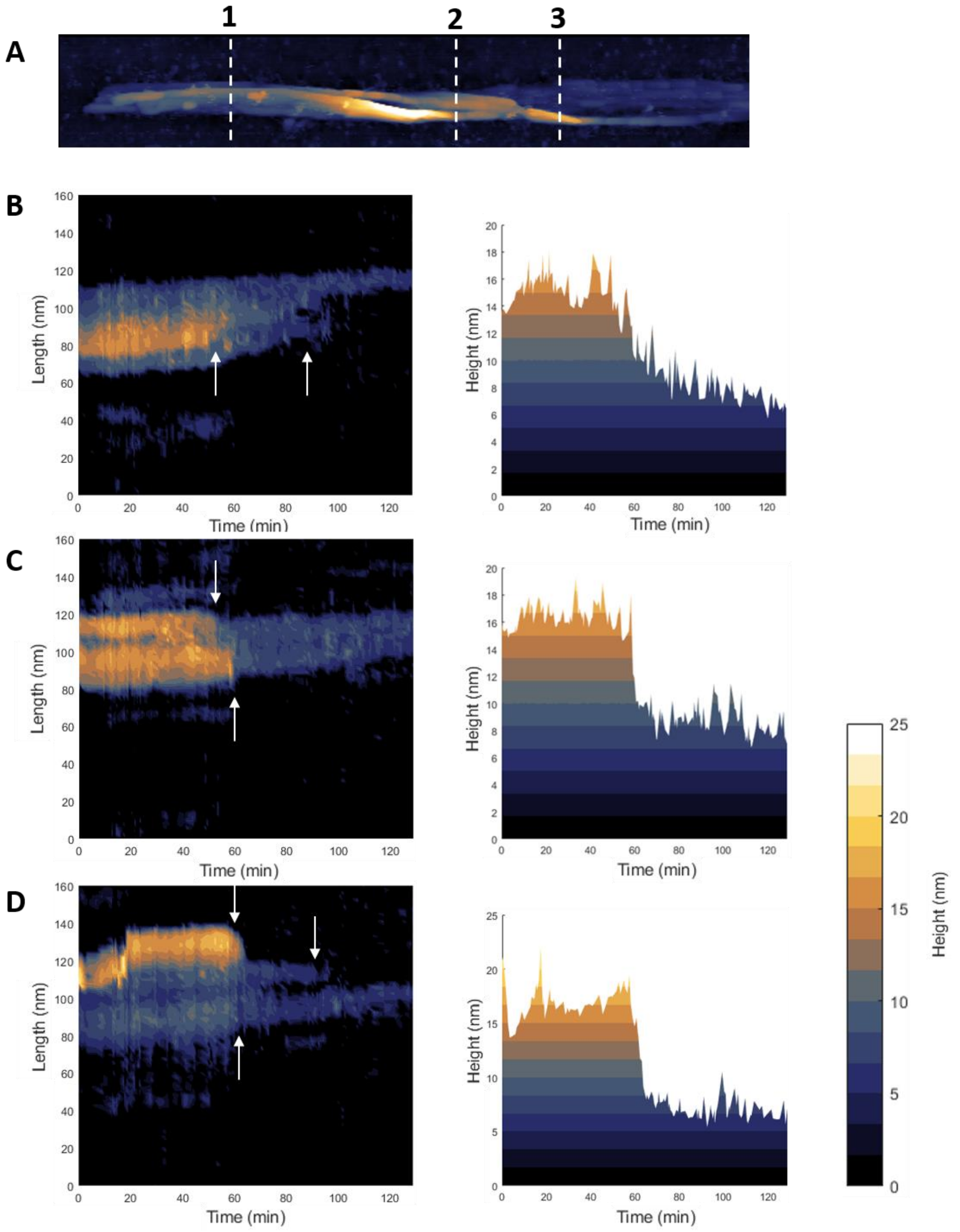

Fig. S15. Additional fiber cross sections showing the same degradation behavior at different positions employed by the cellulosome. (**A**) AFM image of starting frame of the cellulose degradation experiment with indicated exemplary investigated cross sections. 2D plot (left) and maximum values plot (right) of section 1 (**B**), 2(**C**) and 3(**D**). All sections across the

fiber bundle exhibit similar suddenly decreasing fiber increments (white arrows) at positions where beforehand no relevant height change occurs, as depicted in Fig. **6E**.

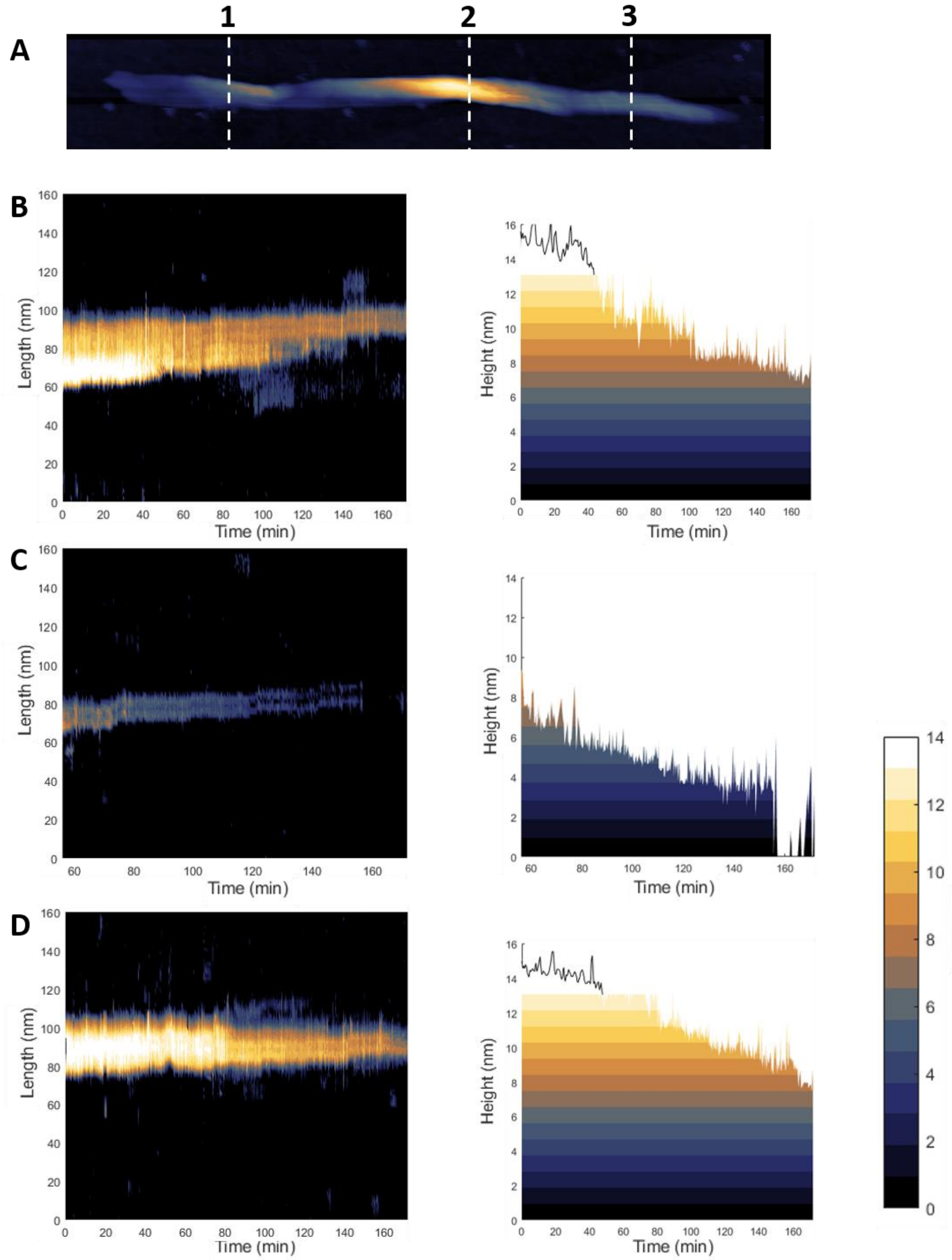

Fig. S16. Additional fiber cross sections showing the same degradation behavior at different positions employed by the dispersed cellulosome. (**A**) AFM image of starting frame of the cellulose degradation experiment with indicated exemplary investigated cross sections. 2D plot (left) and maximum values plot (right) of section 1 (**B**), 2(**C**) and 3(**D**). Section 1 and 3 exhibit similar continuously decreasing height as already seen for the dispersed cellulases.

Also in section 2, after the instable fiber on top is torn away (after 60 min), continuous thinning behavior can be seen until the fiber is degraded completely.

Movie S1: Fragmentation of bacterial cellulose fibers by the cellulosome.

The real time observation of the degradation of bacterial cellulose by the addition of 20 µL buffer solution containing cellulosomes (15 µg/mL). Initially an area of 1200 × 170 nm was observed, later it was reduced to 1000 × 160 nm. Orange dotted circles indicate cellulosomes, rectangles interesting degradation incidents. As prominent examples, the degradations shown at the 29 min, 54 min and 1h 18 min mark show the typical event where the appearance of a cellulosome is immidiatley followed by the disappearance of a fiber segment. Image acquisition rate and resolution was varied from 1.3 -4 frames/min and 1-2 nm/pixel, respectively. Scale bar, time stamps and false color scale are included in the video.

Movie S2: Disruption of big fiber part caused by the cellulosome and the AFM tip.

Real time observation of the severe consequence of the fragmentation style of the cellulosome combined with the AFM tip by addition of 100 µL buffer solution containing cellulosomes (15 µg/mL). The movie displays a cropped region of 930 × 490 nm of the initially measured 1 × 1 µm area. Orange dotted circles indicate cellulosomes, rectangles interesting degradation incidents, orange arrows indicate regions where the cellulosome cuts a fiber part and white arrows indicate influence of the AFM tip. At the 2 min mark the destabilization of a small fiber fragment through cuts can be seen, followed by its disappearance. At the 12 min and 20 min mark it can be seen, that the AFM tip displaces a big part of the fiber after cellulosomes have cut it.

Image acquisition rate and resolution were 0.9 frames/min and 2 nm/pixel, respectively. Scale bar, time stamps and false color scale are included in the video.

Movie S3: Peeling of bacterial cellulose by dispersed cellulases.

Real time observation of the degradation of bacterial cellulose by the addition of 100 µL buffer solution containing the free cellulase mix (15 µg/mL). The movie displays a cropped region of 1160 × 155 nm of the initially measured 1 × 1 µm area. Green dotted circles indicate cellulases, rectangles interesting degradation incidents and green arrows track fiber ends during degradation. Throughout the sequence, the continuous "peeling off" of the outer elementary fibrils can be observed. Stating at the 39 min and 1h 52 min mark, the degradation of two different fibrils occur in opposite directions. At the 2 h mark it can be seen, that the degradation starts at an internal defect.

Image acquisition rate and resolution were 0.6 frames/min and 2 nm/pixel, respectively. Scale bar, time stamps and false color scale are included in the video.

Movie S4: Thinning of bacterial cellulose by dispersed cellulases.

Real time observation of the degradation of bacterial cellulose by the addition of 20 µL buffer solution containing free cellulase mix (15 µg/mL). A scanning area of 400 × 78 nm was observed. Green rectangles indicate interesting degradation incidents and green arrows track fiber ends during degradation. The substantial style of thinning of the dispersed cellulases can be seen throughout the sequence. At the 1 h 35 min mark the degradation start at an internal defect can be seen.

Image acquisition rate and resolution were 0.5 frames/s and 1.5 nm/pixel, respectively. Scale bar, time stamps and false color scale are included in the video.

Movie S5: Longitudinal degradation of bacterial cellulose by the disassembled cellulosome.

Real time observation of the degradation of bacterial cellulose by the addition of 20 µL buffer solution containing disassembled cellulosomes (64 µg/mL). An area of 1000 × 500 nm was observed. Purple dotted circles indicate disassembled cellulosome enzymes, rectangles interesting incidents of degradation and arrows track fiber ends during degradation. At the 15 min, 21 min and 1 h 3 min mark the disassembled cellulosome degrades a single fibril longitudinally, similarly to the dispersed cellulases.

Image acquisition rate and resolution were 1.1 frames/min and 2 nm/pixel, respectively. Scale bar, time stamps and false color scale are included in the video.

Movie S6: Thinning behavior of the disassembled cellulosome on bacterial cellulose.

Real time observation of the degradation of bacterial cellulose by the addition of 20 µL buffer solution containing disassembled cellulosomes (64 µg/mL). An area of 1000 × 160 nm was observed. Purple dotted circles indicate disassembled cellulosome enzymes, rectangles interesting incidents of degradation and arrows track fiber ends during degradation. At the 27 min, 51 min and 1 h 41 min mark the degradation of small fiber fragments by continuous thinning can be seen. At the 2 h 29 min mark, the start of the longitudinal degradation at an internal fiber defect can be seen.

Image acquisition rate and resolution were 3 frames/min and 2 nm/pixel, respectively. Scale bar, time stamps and false color scale are included in the video.

References

1. L. Segal, J. J. Creely, A. E. Martin, C. M. Conrad, An empirical method for estimating the degree of crystallinity of native cellulose using the X-ray diffractometer. *Text. Res. J.* **29**, 786–794 (1959).
2. M. Wada, T. Okano, J. Sugiyama, Synchrotron-radiated X-ray and neutron diffraction study of native cellulose. *Cellulose* **4**, 221–232 (1997).
3. E. I. Kashcheyeva, E. K. Gladysheva, E. A. Skiba, V. V. Budaeva, A study of properties and enzymatic hydrolysis of bacterial cellulose. *Cellulose* **26**, 2255–2265 (2019).
4. R. J. Moon, A. Martini, J. Nairn, J. Simonsen, J. Youngblood, Cellulose nanomaterials review: structure, properties and nanocomposites. *Chem. Soc. Rev.* **40**, 3941–3994 (2011).
5. J. D. Sugar, A. W. Cummings, B. W. Jacobs, D. B. Robinson, A free MATLAB script for spatial drift correction. *Micros. Today* **22**, 40–47 (2014).
6. R. García, J. Tamayo, M. Calleja, F. García, Phase contrast in tapping-mode scanning forcemicroscopy. *Appl. Phys. A Mater. Sci. Process.* **66**, 309–312 (1998).
7. D. Nečas, P. Klapetek, Gwyddion: an open-source software for SPM data analysis. *Open Phys.* **10**, 181–188 (2012).
8. M. G. Resch, B. S. Donohoe, J. O. Baker, S. R. Decker, E. A. Bayer, G. T. Beckham, M. E. Himmel, Fungal cellulases and complexed cellulosomal enzymes exhibit synergistic mechanisms in cellulose deconstruction. *Energy Environ. Sci.* **6**, 1858 (2013).
9. R. Lamed, E. Bayer, Cellulosomes from *Clostridium thermocellum*. *Methods Enzymol.* **160**, 472–482 (1988).

10. M. Eibinger, T. Ganner, H. Plank, B. Nidetzky, A biological nanomachine at work: watching the cellulosome degrade crystalline cellulose. *ACS Cent. Sci.* **6**, 739–746 (2020).
11. N. D. Gold, V. J. J. Martin, Global view of the *Clostridium thermocellum* cellulosome revealed by quantitative proteomic analysis. *J. Bacteriol.* **189**, 6787–95 (2007).
12. E. Morag, S. Yaron, R. Lamed, R. Kenig, Y. Shoham, E. A. Bayer, Dissociation of the cellulosome of Clostridium thermocellum under nondenaturing conditions. *J. Biotechnol.* **51**, 235–242 (1996).
13. H. Esterbauer, W. Steiner, I. Labudova, A. Hermann, M. Hayna, Production of *Trichoderma* cellulase in laboratory and pilot scale. *Bioresour. Technol.* **36**, 51–65 (1991).
14. M. Eibinger, P. Bubner, T. Ganner, H. Plank, B. Nidetzky, Surface structural dynamics of enzymatic cellulose degradation, revealed by combined kinetic and atomic force microscopy studies. *FEBS J.* **281**, 275–290 (2014).
15. M. Eibinger, J. Sattelkow, T. Ganner, H. Plank, B. Nidetzky, Single-molecule study of oxidative enzymatic deconstruction of cellulose. *Nat. Commun.* **8**, 894 (2017).
16. M. Lemos, J. Teixeira, M. Mota, F. Gama, A simple method to separate cellulose-binding domains of fungal cellulases after digestion by a protease. *Biotechnol. Lett.* **22**, 703–707 (2000).
17. D. Kumar, G. S. Murthy, Stochastic molecular model of enzymatic hydrolysis of cellulose for ethanol production. *Biotechnol. Biofuels* **6**, 63 (2013).
18. Y. Hu, J. M. Catchmark, Integration of cellulases into bacterial cellulose: toward bioabsorbable cellulose composites. *J. Biomed. Mater. Res. Part B Appl. Biomater.* **97B**, 114–123 (2011).
19. E. Bayer, R. Lamed, Affinity digestion for the near-total recovery of purified cellulosome from *Clostridium thermocellum*. *Enzyme Microb. Technol.* **14**, 289–292

(1992).

20. S. Bauer, A. B. Ibáñez, Rapid determination of cellulose. *Biotechnol. Bioeng.* **111**, 2355–2357 (2014).
